# Controlling supramolecular filament chirality of hydrogel by co-assembly of enantiomeric aromatic peptides

**DOI:** 10.1186/s12951-022-01285-0

**Published:** 2022-02-10

**Authors:** Xuejiao Yang, Honglei Lu, Yinghua Tao, Hongyue Zhang, Huaimin Wang

**Affiliations:** 1 Key Laboratory of Precise Synthesis of Functional Molecules of Zhejiang Province, School of Science, Westlake University, Hangzhou, China; 2 Institute of Natural Sciences, Westlake Institute for Advanced Study, 18 Shilongshan Road, Hangzhou, 310024 Zhejiang Province China; 3 Westlake Laboratory of Life Sciences and Biomedicine, School of Life Sciences, Westlake University, Hangzhou, Zhejiang China

**Keywords:** Hydrogel, Self-assembly, Peptide, Supramolecular chirality, Cell culture

## Abstract

Supramolecular chirality plays an indispensable role in living and synthetic systems. However, the generation and control of filament chirality in the supramolecular hydrogel of short peptides remains challenging. In this work, as the first example, we report that the heterodimerization of the enantiomeric mixture controls the alignment, chirality, and stiffness of fibrous hydrogels formed by aromatic building blocks. The properties of the resulting racemic hydrogel could not be achieved by either pure enantiomer. Cryo-EM images indicate that the mixture of L and D enantiomers forms chiral nanofibers, the percentage of which can be readily controlled through stoichiometric co-assembly of heterochiral enantiomers. 2D NOESY NMR and diffusion-ordered NMR spectroscopy reveal that heterodimerization of enantiomers plays a crucial role in the formation of chiral nanofibers. Further mechanistic studies unravel the mechanism of supramolecular chirality formation in this two-component system. Molecular dynamics simulations confirm that the intermolecular hydrogen bond and π–π interaction of heterodimers play important roles in forming a chiral hydrogel. Furthermore, regulation of the adhesion and morphology of mammalian cells is achieved by tuning the relative ratio of L and D enantiomers at the same concentration. This work illustrates a novel strategy to control the supramolecular chirality of aromatic peptide hydrogels for materials science.

**Graphical Abstract:**

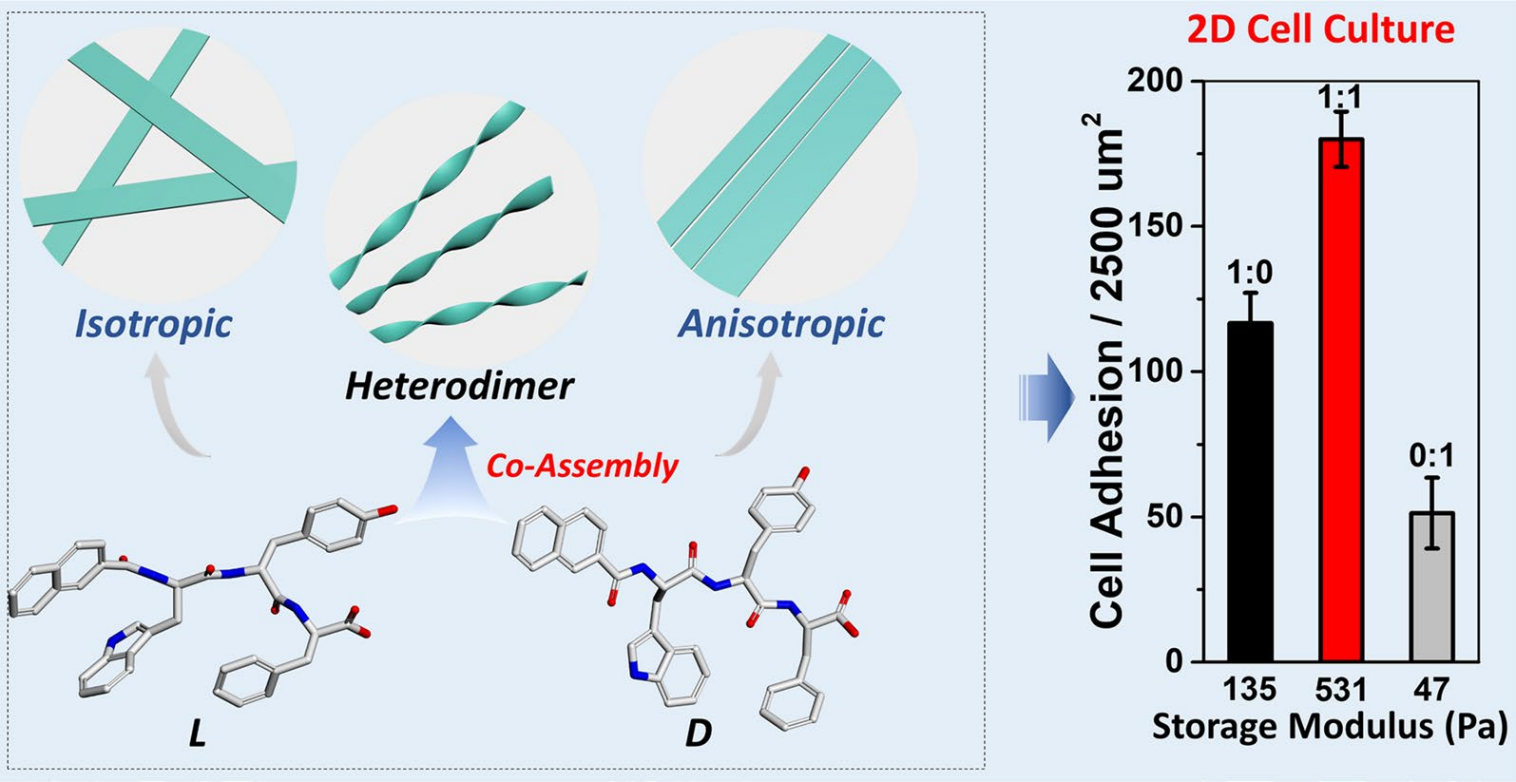

**Supplementary Information:**

The online version contains supplementary material available at 10.1186/s12951-022-01285-0.

## Introduction

Supramolecular chirality is a critical feature in living and synthetic systems. Remarkable examples are the double-helical DNA which stores genetic information and the triple-helical collagen fibers that constitute the primary structural element of the extracellular matrix (ECM) [1, 2]. The elucidation of atomic-resolution structures of functional proteins also revealed that supramolecular chirality prevails in almost all signal transduction processes [3]. For example, the assembly of the death fold domains, pyrin domain, and the caspase recruitment domain into helical filaments amplifies caspase activation to generate a sufficient cellular response [4]. The increasing realization of the importance of supramolecular chirality [5] in biological activities opens up opportunities for developing soft materials (e.g., hydrogel) for chiral recognition [6], drug delivery [7, 8], catalysis [5, 9], sensing [10], and 3D cell culture [11].

Resembling the properties of native ECM, the supramolecular hydrogel has been attracting more and more attention in different disciplines, including chemistry, materials, and biology. To construct hydrogel with chiral supramolecular structures, self-assembly through non-covalent interactions is an efficient strategy [5, 7, 12–14]. A few approaches have been investigated to induce the chirality of nanofibers within the hydrogel, including metal ions, pH, achiral additives, and temperature. For example, Feng group demonstrated that metal-ion could mediate the supramolecular chirality of small molecules to form hydrogel for cell adhesion [15]. Zhao and coworkers employed achiral bis(pyridinyl) derivatives to tune the handedness of nanofibers in C2 phenylalanine-based hydrogel [16]. Jiang and Yang’s groups found that enzyme instructed self-assembly of the peptide could induce the formation of right-handed nanofibrous hydrogel to modulate the immune response [17]. Although these approaches are relatively successful and practical, the requirement of external metal ions, enzymes, and achiral inducers still limits their application. Another recent finding revealed that molecular chirality could affect the self-assembly of short amphiphilic peptides and dictate the final morphological handedness by non-covalent interactions [18–24]. For example, Adams and Serpell have described a single amino acid variation could govern the assemblies helicity of dipeptide [20]. Marchesan and co-workers have shown that amino acid’s steric configuration plays a key role in the self-assembly of unprotected tripeptides and dipeptides [21]. Adams and co-workers also demonstrated that the properties of gels could be tuned simply by varying the chirality of dipeptide [22]. Cui and Stupp groups have described that the sequence of amino acids in isomeric tetrapeptide with hydrophobic alkyl could dictate the final 1D nanostructures [25]. Despite these advancements, it remains challenging to control the supramolecular chirality of nanofibrous hydrogel with tunable alignment [26].

Herein, we report that controlling the intermolecular interaction of enantiomeric tripeptide with aromatic modification affords a chiral nanofibrous hydrogel with tunable alignment, which could not be achieved by either pure enantiomer. We found that L enantiomeric peptide forms an isotropic hollow nanofibrous hydrogel, while its corresponding D isomer forms the anisotropic hydrogel with aligned hollow nanofibers. The mixture of the L and D isomers co-assemble to form chiral nanofibers with controlled alignment and stiffness. Simply tuning the ratio of L and D enantiomers, we can control the ratio of chiral nanofibers in the molecular hydrogel. Detailed mechanistic studies indicated that the chiral nanofibers consist of the heterodimer of L and D enantiomers through non-covalent interactions. Molecular dynamics simulations confirm that intermolecular hydrogen bonding and π–π stacking play a key role in forming heterodimers. Moreover, we also demonstrate the good biocompatibility of the hydrogels by 2D and 3D cell culture. This work illustrates a new and efficient strategy to generate supramolecular chiral nanofibers with tunable alignment and stiffness, which could be used to create programmable niches for directing cell functions.

## Results and discussion

**Fig. 1:**
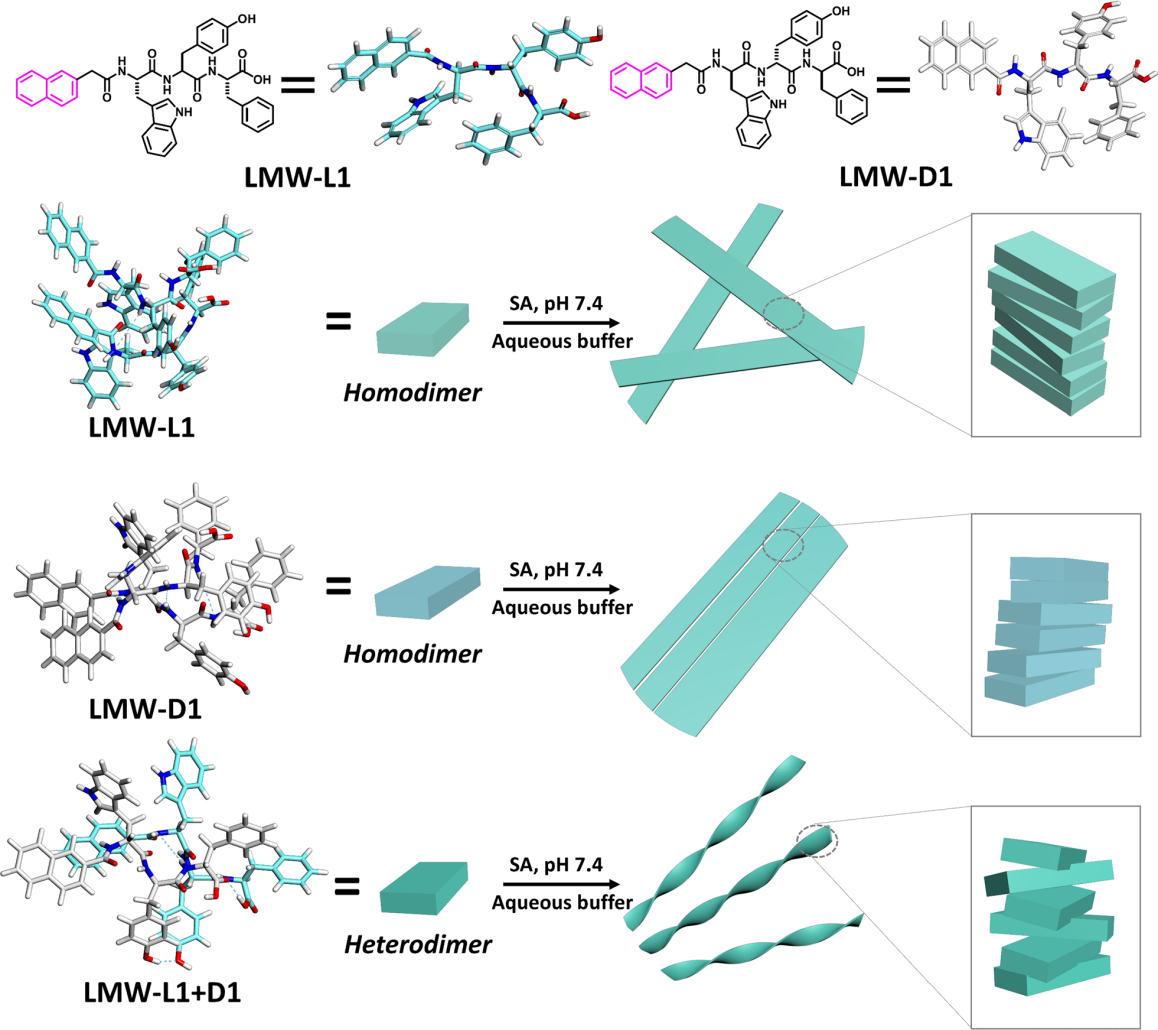
Chemical structures of LMW-L1 and LMW-D1, and the illustration of self-assembly mechanisms for three kinds of low molecular weight (LMW) hydrogels that formed at pH 7.4 aqueous solution. Homodimerization of LMW-L1 or LMW-D1 forms hydrogel with isotropic or anisotropic nanofibers, while the mixture of enantiomer LMW-L1 and LMW-D1 co-assembles to form chiral nanofibrous hydrogel consisting of heterodimers. The cyan dotted line represents hydrogen bonding

As shown in Fig. 1, we designed low molecular weight (LMW) building blocks based on the following rationales: (i) Tryptophan-tyrosine (WY) are favorable amino acid composition at the protein–protein interface, which engage in hydrogen bonding and π–π stacking [27–29]; (ii) (Naphthalene-2-ly) acetyl group (Nap) provides strong intermolecular aromatic-aromatic interactions to enable self-assembly and maintain the secondary structure of short peptides [30]; (iii) Phenylalanine is a well-known amino acid to form amyloid-like deposits [31]. Therefore, we synthesized L (LMW-L1) and D enantiomer (LMW-D1) of short peptide 2-(naphthalene-2-yl)acetic-Trp-Tyr-Phe using solid phase peptide synthesis (Additional file 1: Scheme S1) using 2-chlorotrityl chloride resin and the corresponding N^α^-Fmoc-protected amino acids with side chains properly protected. We used high performance liquid chromatography (HPLC) to purify all the molecules and characterized them by NMR and LC–MS (Additional file 1: Figs. S2, S3, S8, S9).

**Fig. 2:**
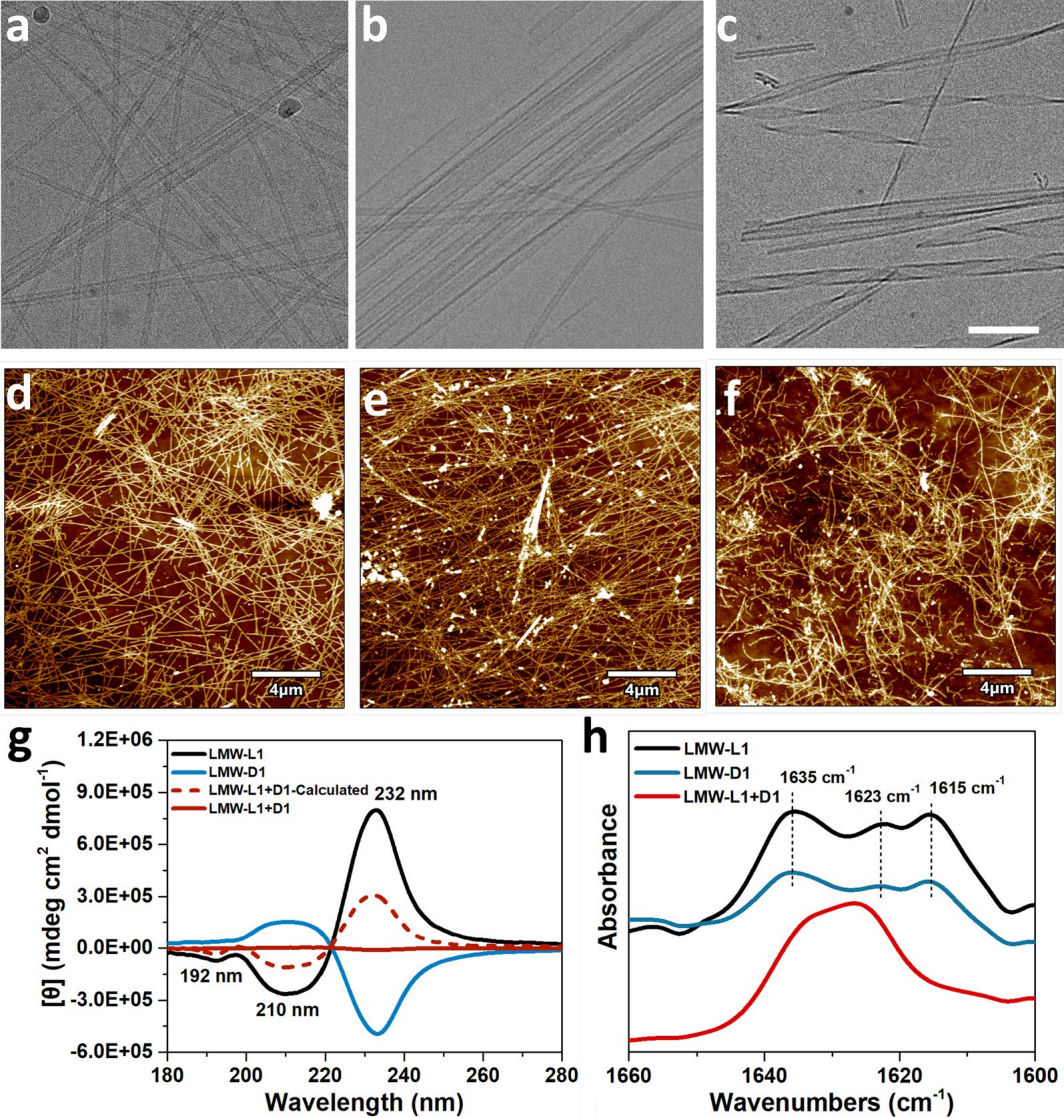
Cryo-EM and AFM images of hydrogels formed by (**a**, **d**) LMW-L1, (**b**, **e**) LMW-D1, (**c**, **f**) equimolar mixture of LMW-L1 and LMW-D1. Scale bar in **a**–**c** is 100 nm. **g** CD and **h** FTIR spectra of hydrogels in **a**–**c**. The concentration of LMW-L1 or LMW-D1 is 0.3 wt%

We first examine the hydrogelation and self-assembly performance of the molecules. We used the heating–cooling strategy to form a hydrogel, a common strategy to induce gelation [32]. After heating at 75 °C for 1 min, the solution of the hydrogelator dissolves completely and then cooling leads to hydrogelation. Dissolving in phosphate buffer (pH 7.4) at 0.3 wt%, LMW-L1 or LMW-D1 forms a translucent hydrogel (Additional file 1: Fig. S14) within 10 min by heating–cooling strategy. Further gelation ability measurement shows that LMW-L1 and LMW-D1 have similar critical gelation concentration (CGC) of 0.2 wt%. Interestingly, the equimolar mixing (0.3 wt %) of enantiomers resulted in rapid gelation within 5 min to form a racemic hydrogel with excellent stability at room temperature for several months. Cryogenic transmission electron microscopy (cryo-TEM) and AFM images indicate that LMW-L1 forms hydrogel consisting of randomly entangled hollow nanofibers with an average diameter and height of 10 nm and 10 nm, respectively. In contrast, the hydrogel of LMW-D1 composes of extraordinarily long arrays of aligned hollow nanofibrous bundles. The average diameter and height is 24 nm and 9 nm, respectively (Fig. 2; Additional file 1: Figs. S15–S17), which differs from recent work showing that L and D enantiomers of the peptide have similar morphologies [18, 33–35]. The equimolar mixture of LMW-L1 and LMW-D1 assembles into supramolecular left-handed nanohelices with a diameter and height of 19 nm and 7 nm (Fig. 2c, f; Additional file 1: Figs. S15–S17), respectively. These results suggest the feasibility of enantiomeric mixing to induce supramolecular chirality, which has not been reported previously. Furthermore, to eliminate the pre-formed nanostructures during the peptide purification before the assembly, we dissolve the LMW-L1 and LMW-D1 in 1,1,1,3,3,3-hexafluoro-2-propanol (HFIP), respectively, after freeze- drying, we perform the co-assembly experiments. The equimolar mixture of LMW-L1 and LMW-D1 could also form nanohelices (Additional file 1: Fig. S18), consistent with the above observations.

To investigate the secondary structure of hydrogels, we employed circular dichroism (CD) and Fourier transform infrared spectroscope (FTIR). The CD spectrum (Fig. 2g) of LMW-L1 exhibits two negative peaks located at 192 nm and 210 nm, which correspond to the π–π* transition and n–π* transition of peptide chromophores, and one positive peak at 232 nm, which could be attributed to the exciton coupling of the naphthalene chromophores.[36] The hydrogel of LMW-D1 shows a mirror symmetry to LMW-L1 hydrogel. The CD spectrum from the equimolar mixture of enantiomers (experimental) is not identical to the simple sum (theoretical) of two single-component spectra (Fig. 2g), suggesting the co-assembly of enantiomers [33, 37]. Furthermore, we recorded the HT data with the measurement of the CD signals (Additional file 1: Fig. S19). We accounted for the contribution of light scattering from the PBS, and the HT data represented the corresponding absorbance of the hydrogels. In the HT spectrum, the hydrogels showed similar absorbance in UV–vis spectra. They exhibit three absorption peaks located around 190 nm, 220–230 nm, and 280–290 nm, corresponding to the π–π* transition, n–π* transition, and the absorbance of naphthalene chromophore [20], respectively. In-situ FTIR spectroscopy shows that the hydrogels formed by LMW-L1 and LMW-D1 exhibit strong amide I stretch at 1615 cm^−1^, 1623 cm^−1^, and 1635 cm^−1^ (Fig. 2h), which corresponding to the β-sheet secondary structure [23]. In contrast, the hydrogel co-assembled by equimolar of LMW-L1 and LMW-D1 exhibit the red shift with two absorption peaks at 1633 cm^−1^ and 1626 cm^−1^, indicating the co-assembly property.

**Fig. 3:**
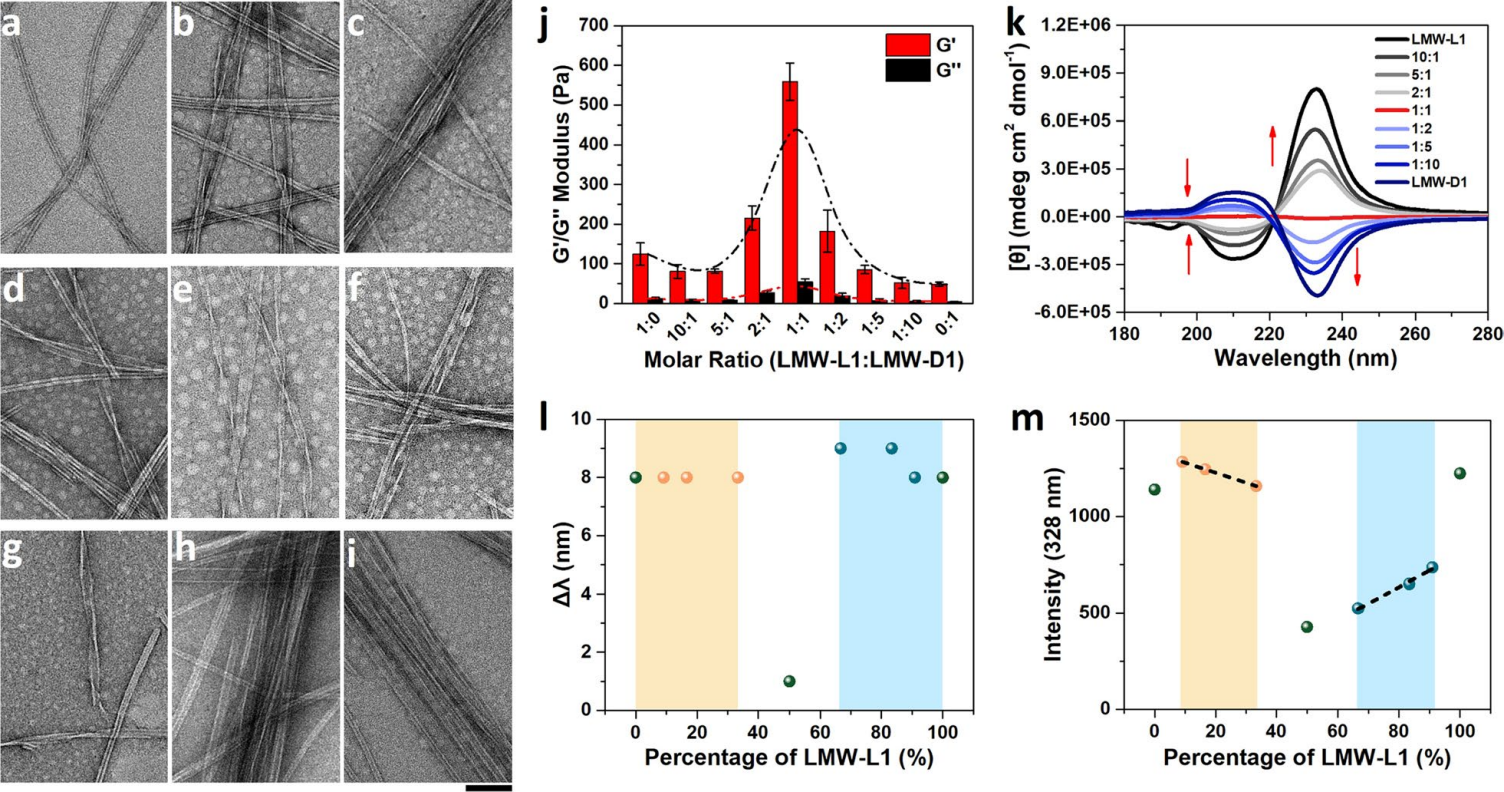
TEM images of hydrogel formed by LMW-L1 and LMW-D1 at a molar ratio of (**a**) 1:0, (**b**) 10:1, (**c**) 5:1, (**d**) 2:1, (**e**) 1:1, (**f**) 1:2, (**g**) 1:5, (**h**) 1:10, and (**i**) 0:1. Scale bar is 100 nm. **j** Rheology results (n = 3) and **k** CD spectra of hydrogels. **l** The wavelength offset in UV–vis spectra. **m** The intensity of 328 nm in fluorescence spectra in dependence of LMW-L1’s percentage

To further investigate the interaction between LMW-L1 and LMW-D1, we examine the self-assembly performance of these enantiomers at various relative ratios by high-resolution TEM. Gelation test indicates that the mixture of LMW-L1 and LMW-D1 at 0.3 wt% forms stable hydrogels despite the different relative concentrations of LMW-L1 and LMW-D1 (Additional file 1: Fig. S14, Table S2). As shown in Fig. 3a–i, supramolecular helical structures gradually developed with the increasing percentage of LMW-D1, and the helical nanofibers constitute almost 100% of nanostructures at the molar ratio of 1:1. Moreover, the hydrogel mainly consists of isotropic nanofibers when LMW-L1 is the major component, while anisotropic nanofibers are formed with the increase of LMW-D1 (Fig. 3a-i; Additional file 1: Fig. S20). These results, together with Cryo-EM experiments of hydrogels, indicate that drying has little effect on the structures in our system [38], further suggesting that the supramolecular chiral fibers are formed by the co-assembly of LMW-L1 and LMW-D1. This observation also demonstrates that simple stoichiometric co-assembly of biomolecular enantiomers is a new strategy to tune the chirality and alignment of nanofibers [26].

We next measure the mechanical properties of the above hydrogels with rheometry. The hydrogel formed by the equimolar mixture of enantiomers exhibits the highest rigidity, over 4 and 11 folds greater than that of the hydrogel formed by LMW-L1 and LMW-D1, respectively (Fig. 3j; Additional file 1: Figs. S21, S22, Table S3). Specifically, we could observe more anisotropic nanofibers in the hydrogel with the increase of amount of LMW-D1, and the mechanical strength of the corresponding hydrogel decreases gradually (Fig. 3j). The storage modulus of hydrogel formed by LMW-D1 reaches the lowest, which is consistent with the observation of Adams et al. in the dipeptide system [22]. Adjusting the enantiomer ratios enables us to modulate the mechanical properties of the supramolecular hydrogels. More specifically, with the increase of LMW-D1, the storage modulus of hydrogels increases first and then decreases, which attained the highest value at the molar ratio of 1:1. However, the values of storage modulus show a little difference with the alternation of molar ratio from 1:0 to 0:1. The average storage modulus is 124.7 ± 28.4 Pa (1:0), 80.7 ± 17.6 Pa (10:1), 81. 6 ± 5.1 Pa (5:1), 215.7 ± 30.3 Pa (2:1), 558.4 ± 46.9 Pa (1:1), 182.4 ± 53.2 Pa (1:2), 85.1 ± 10.6 Pa (1:5), 51.6 ± 14.1 Pa (1:10), and 48.2 ± 2.0 Pa (0:1), respectively. The molar ratio-dependent CD studies indicate that the signal at 232 nm gradually decreases with the increase of LMW-D1’s content. The CD signal approaches almost the baseline when the molar ratio is 1:1, where the inter-chromophore orientation is chiral but racemic (Fig. 3k), revealing the co-assembly state in the supramolecular chirality. In the UV–vis spectra, the hydrogels at different molar ratios of LMW-L1 and LMW-D1 show a redshift compared to monomers (Additional file 1: Fig. S23a), indicating the formation of aggregates. However, the hydrogel formed by equimolar ratio of enantiomers only shows a little redshift of 1 nm (Fig. 3l), indicating the interaction of racemic inter-chromophores. The emission spectra (Fig. 3m) of hydrogels suggest the strong interaction between LMW-L1 and LMW-D1 within chiral nanofibers. For example, the weakest emission at 328 nm of an equal molar mixture of enantiomers suggests that the aggregation suppresses most fluorescence (Additional file 1: Fig. S23c; Fig. 3m), most likely due to the energy transfer from the monomeric to the excimeric Nap group [39].

**Fig. 4:**
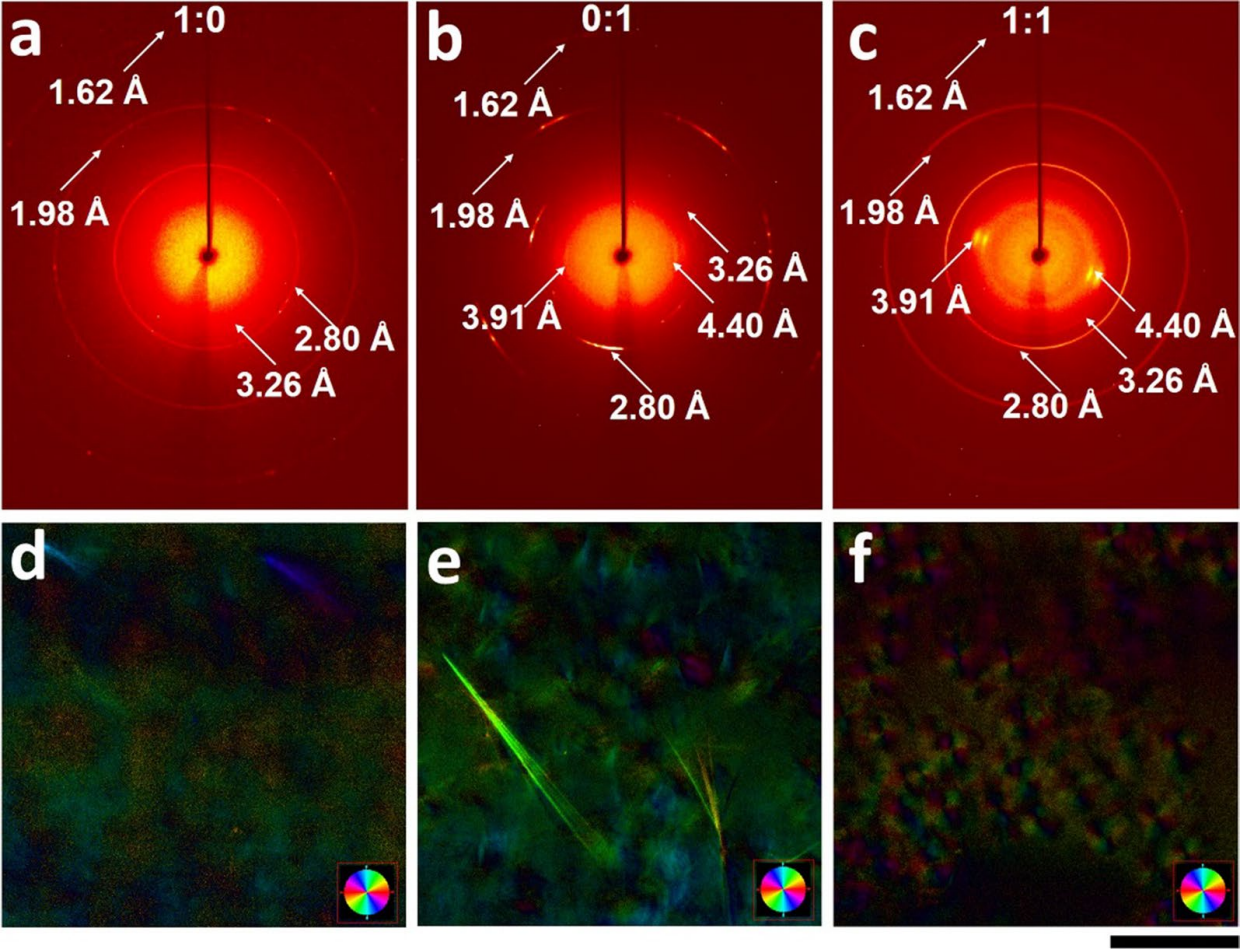
**a**–**c** In situ WAXS patterns and (**d**–**f**) birefringence of hydrogels formed by (**a**, **d**) LMW-L1, (**b**, **e**) LMW-D1, and (**c**, **f**) equimolar mixture of two enantiomers. Scale bar is 100 μm

To study the molecular packing of LMWs within nanofibrous hydrogels, in situ wide angle X-ray diffraction (WAXS) experiments were performed. We observe the uniform diffraction rings corresponding to the *d* spacing of 1.62 Å, 1.98 Å, 2.80 Å, and 3.26 Å for LMW-L1 (Fig. 4a). The diffractions of 1.62 Å and 1.98 Å indicate the periodic distance between adjacent peptide and the 2.80 Å and 3.26 Å diffraction attribute to the periodic intermolecular hydrogen bonding and π–π stacking between adjacent peptide segments within the β-sheet secondary structure. With the increasing percentage of LMW-D1, there appear two orientational signals corresponding to the *d* spacing of 3.91 Å and 4.40 Å (Additional file 1: Fig. S24), indicating the orientational order of nanofibers within the hydrogel and the formation of well-aligned nanofibers is driven by strong π–π interaction. Specifically, the hydrogel co-assembled by an equimolar mixture of LMW-L1 and LMW-D1 exhibits the consecutive and strong diffraction rings, suggesting the homogeneity of nanostructures in the hydrogels (Fig. 4b). However, the hydrogel of LMW-D1 shows crystal-like discrete diffraction rings (Fig. 4c), likely due to the rigid anisotropic nanofibers within the hydrogels [40]. Furthermore, the hydrogel of LMW-D1 also exhibits the birefringent domains (Fig. 4e), while the other two kinds of hydrogels appear entire isotropic with no birefringence (Fig. 4d, f). These results further support that the simple mixture of enantiomers could control the supramolecular chirality and alignment of nanofibers.

**Fig. 5:**
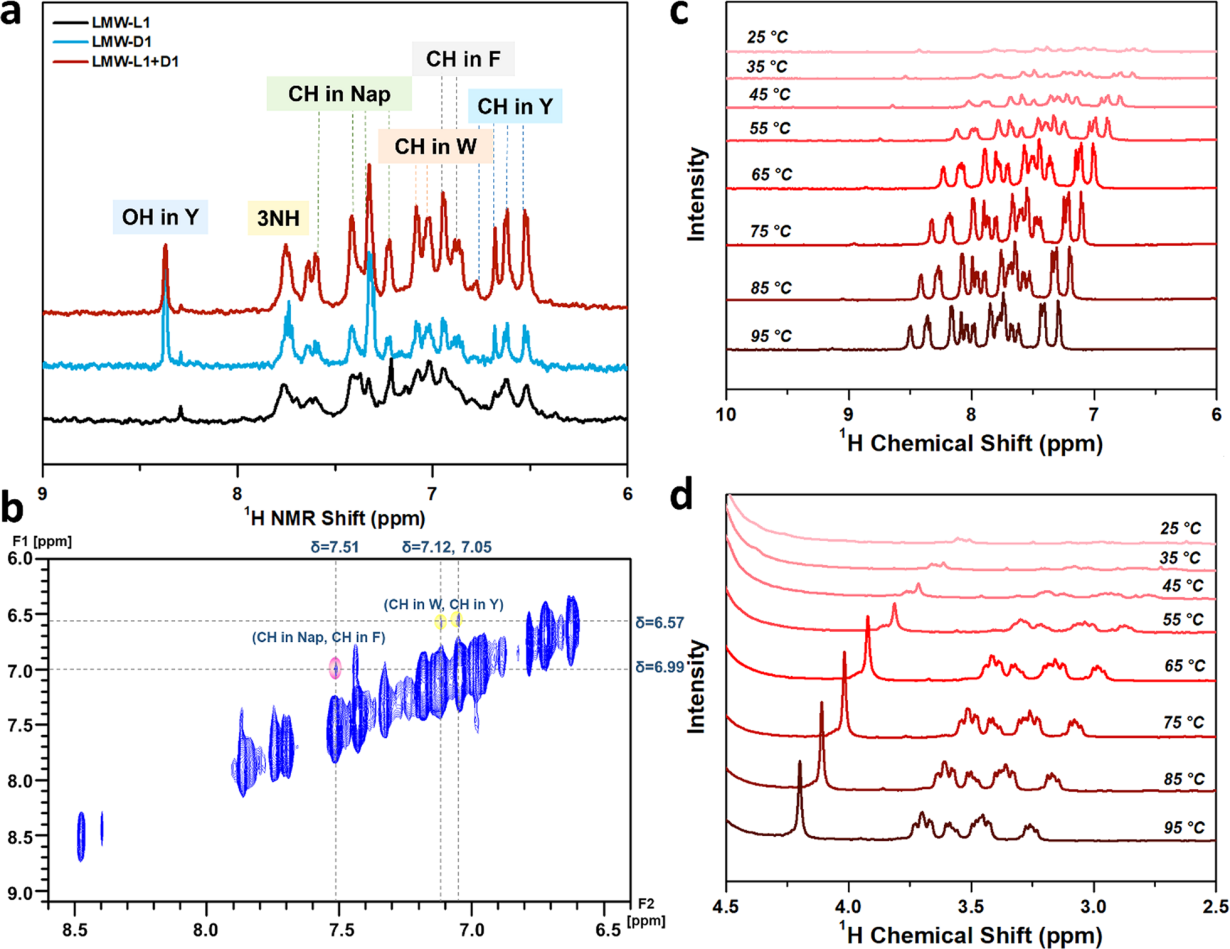
**a** ^1^H NMR spectrum of nanostructures formed by LMW-L1 and LMW-D1 in deuterated PBS. **b** 2D NOESY spectrum of nanostructures formed by LMW-L1 and LMW-D1 at a molar ratio of 1:1. Close contacts are showing in colored circles. **c**, **d** ^1^H NMR spectrum of nanostructures formed by equal molar ratio of LMW-L1 and LMW-D1 in deuterated PBS at different temperature from 25 ºC to 95 ºC, the ^1^H NMR spectrum of nanostructures was detected every 10 ºC. The total concentration of LMW-L1 and LMW-D1 is 0.1 wt%.

We next use NMR further to investigate the specific molecular packing within the assembled nanostructures. Compared with the monomer in *d*_*6*_-DMSO, ^1^H NMR signals of LMW-L1 and LMW-D1 in D_2_O move to the lower field with agminated and broadened peaks, revealing the formation of assemblies (Fig. 5a; Additional file 1: Fig. S25) [41]. The aggregation of peaks within the aromatic region is more obvious. Together with structure–activity relationship studies, these results suggest the importance of the aromatic-aromatic interaction in the formation of nanostructures (Additional file 1: Figs. S4–S7, S10–S13, S26, S27). Moreover, the ^1^H NMR signal of the enantiomeric mixture is not the simple sum of the LMW-L1 and LMW-D1 spectra, further demonstrating that the chiral nanostructures are formed through co-assembly. Diffusion-ordered NMR spectroscopy (DOSY) reveals that the diffusion coefficient of hydrogel formed by LMW-L1, LMW-D1, and their equimolar mixture is 2.213 × 10^–10^ m^2^ s^−1^, 2.529 × 10^–10^ m^2^ s^−1^, and 2.275 × 10^–10^ m^2^ s^−1^ (Additional file 1: Fig. S28–30), respectively. These results suggest that LMW-L1 and enantiomeric mixture form dimers and trimers, while the nanostructure formed by LMW-D1 consists of monomers and dimers [42]. Furthermore, we used 2D nuclear Overhauser effect spectroscopy (2D NOESY) to investigate the H–H correlation (Additional file 1: Fig. S31–S33). Close contacts (< 3 Å) are observed between the CH in Nap (δ = 7.43 ppm) and CH_2_ in F and W (δ = 2.94, 3.55 ppm). We also find the close contacts between CH in Nap (δ = 7.51 ppm) and F (δ = 6.99 ppm), CH in W (δ = 7.12, 7.05 ppm) and Y (δ = 6.61, 6.58 ppm) (Fig. 5b). The correlation in spatiality of Nap with F, Nap with W, and W with Y attribute to the co-assembly of LMW-L1 and LMW-D1 (Fig. 1). Furthermore, the temperature-dependent ^1^H NMR spectra show that the NMR signals moved to the lower field when the temperature increased from 25 ℃ to 95 ℃, and the shape of signals changed. The results indicate that the aggregated state of LMW-L1 and LMW-D1 is dissociating. However, the aggregated state of the peaks could not be disturbed totally even at 95 ℃ (5 min), indicating the mixtures still hold the co-assembly state, revealing the robust stability of nanostructure co-assembled by LMW-L1 and LMW-D1 (Fig. 5c, d).

**Fig. 6:**
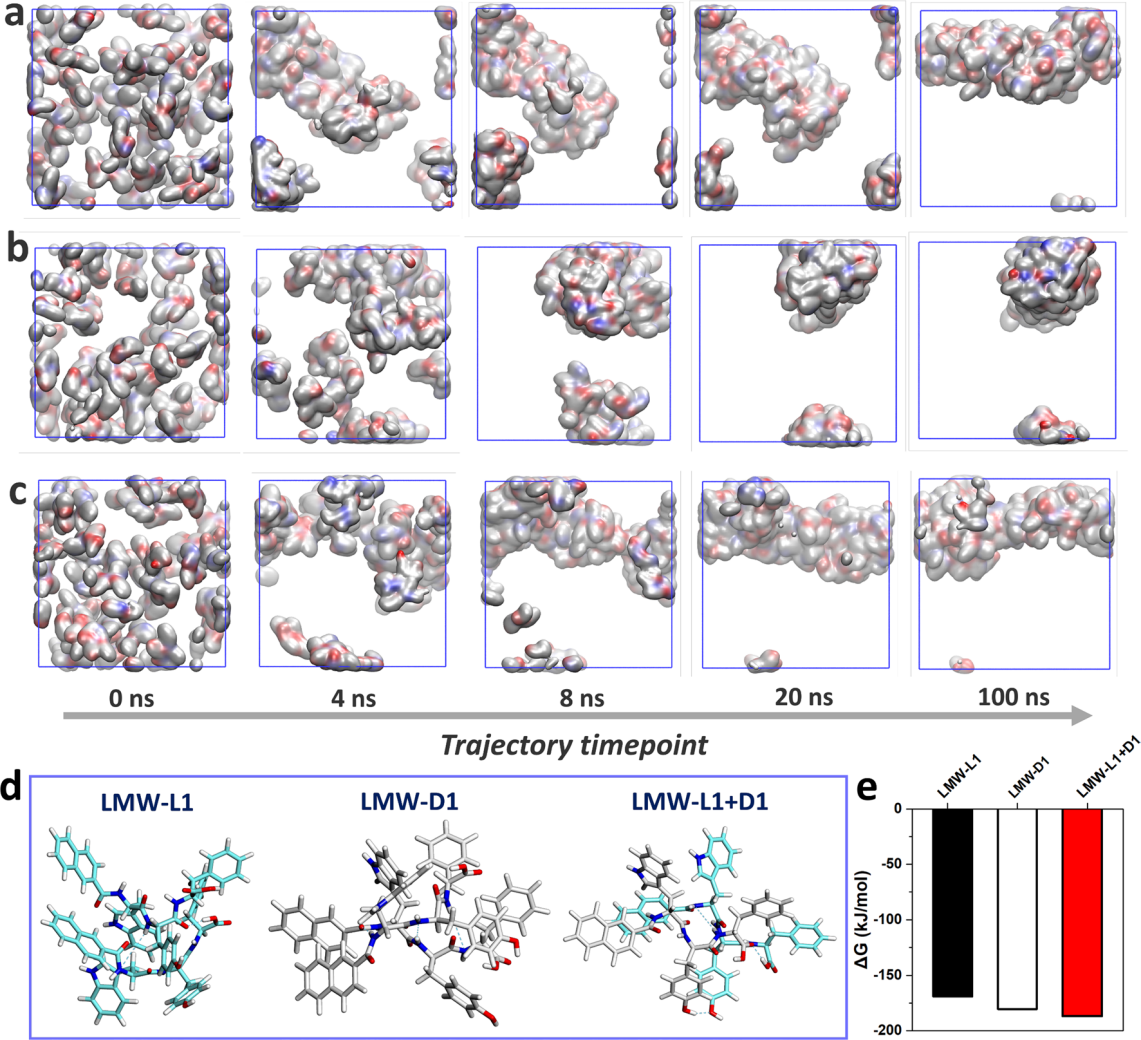
Spatiotemporal evolution of the simulated nanostructures formed by (**a**) LMW-L1, (**b**) LMW-D1, and (**c**) equimolar mixture of two enantiomers at different timepoint. **d** Optimized structures of homodimers and heterodimers via MD calculation. **e** Relative total energies for homodimers and heterodimers. The cyan dotted line represents the hydrogen bonding

We next perform molecular dynamics (MD) simulations to investigate the molecular interactions during the self-assembly processes. The MD simulations were performed by the Gromacs software package [43] under the Amber99SB-ILDN force field [44]. Specifically, 50 LMW-L1 and LMW-D1 molecules at a ratio of 1:0, 1:1, and 0:1 are randomly added into three indicate 8 nm periodic boundary condition (PBC) boxes, separately, then the boxes are filled with TIP3P water molecules [45]. We performed 5000 steepest descent steps and 5000 conjugate gradient minimization steps to achieve energy minimization of the system. Then a 200 ps restraint simulation at a time step of 1 fs was performed in the NPT ensemble to make water molecules relax, followed by production simulation of 100 ns in the NPT ensemble at a temperature of 398.15 K. We used the Particle Mesh Ewald (PME) method [46] to treat long-range electrostatic interactions with a cut-off value of 1.0 nm and then analyzed the MD trajectory [47]. The peptides adopt the random arrangement at 0 ns. However, the nanostructures formed by LMW-L1 and an equimolar mixture of LMW-L1 and LMW-D1 could rapidly form aggregates within 4 ns (Fig. 6a–c), which is faster than the nanostructure formed by LMW-D1. The three kinds of aggregates tend to stabilize after 8 ns and 20 ns. Moreover, the state of equimolar mixture of LMW-L1 and LMW-D1 at 20 ns is more similar to the final state (100 ns), which is different from the aggregates formed by LMW-L1 and LMW-D1, respectively. Finally, LMW-L1 and LMW-D1 could form cylinder-like aggregates, and the diameter of LMW-D1 is larger than LMW-L1, consisting with the observation by TEM. The cylinder-like aggregates formed by an equimolar mixture of LMW-L1 and LMW-D1 show the nonuniform diameter, similar to helix-like nanostructures (Additional file 1: Fig. S34). Furthermore, we perform the MD calculations on homodimers and heterodimers formed by LMW-L1 and LMW-D1. We could observe the intermolecular hydrogen bonding between two N–H, C=O and COOH, as well as π–π stacking between Nap in the homodimers formed by LMW-L1 (Fig. 6d). In the homodimers formed by LMW-D1, hydrogen bonds between two N–H, N–H and C=O, as well as π–π stacking, stabilize the structure. Combining with the 2D DOSY results, the difference between intermolecular interactions and aggregate state may contribute to the nanofibers at different morphology formed by LMW-L1 and LMW-D1. However, the hydrogen bonding between two O–H and COOH appeared in the heterodimers formed by LMW-L1 and LMW-D1, which have never been observed in homodimers. Meanwhile, we hardly observe the direct interaction between LMW-L1 and LMW-D1 themselves. In addition, the relative total energies for LMW-L1-LMW-L1 and LMW-D1-LMW-D1 are − 169.1 kJ/mol and − 180.2 kJ/mol, respectively, while the relative total energy for heterodimer of LMW-L1 and LMW-D1 is − 186.8 kJ/mol (Fig. 6e), indicating that the LMW-L1/LMW-D1 hetero-interactions are more favorable than the homo-interactions. The MD simulations also show the strong π–π stacking between Nap (LMW-L1) and F (LMW-D1), and Nap (LMW-D1) and W (LMW-L1) (Fig. 6c) in chiral nanofiber, agreeing with the 2D NOESY results. These results suggest that the intramolecular hydrogen bonding and π–π stacking between Nap promote the self-assembly of LMW-L1 and LMW-D1, respectively.

**Fig. 7:**
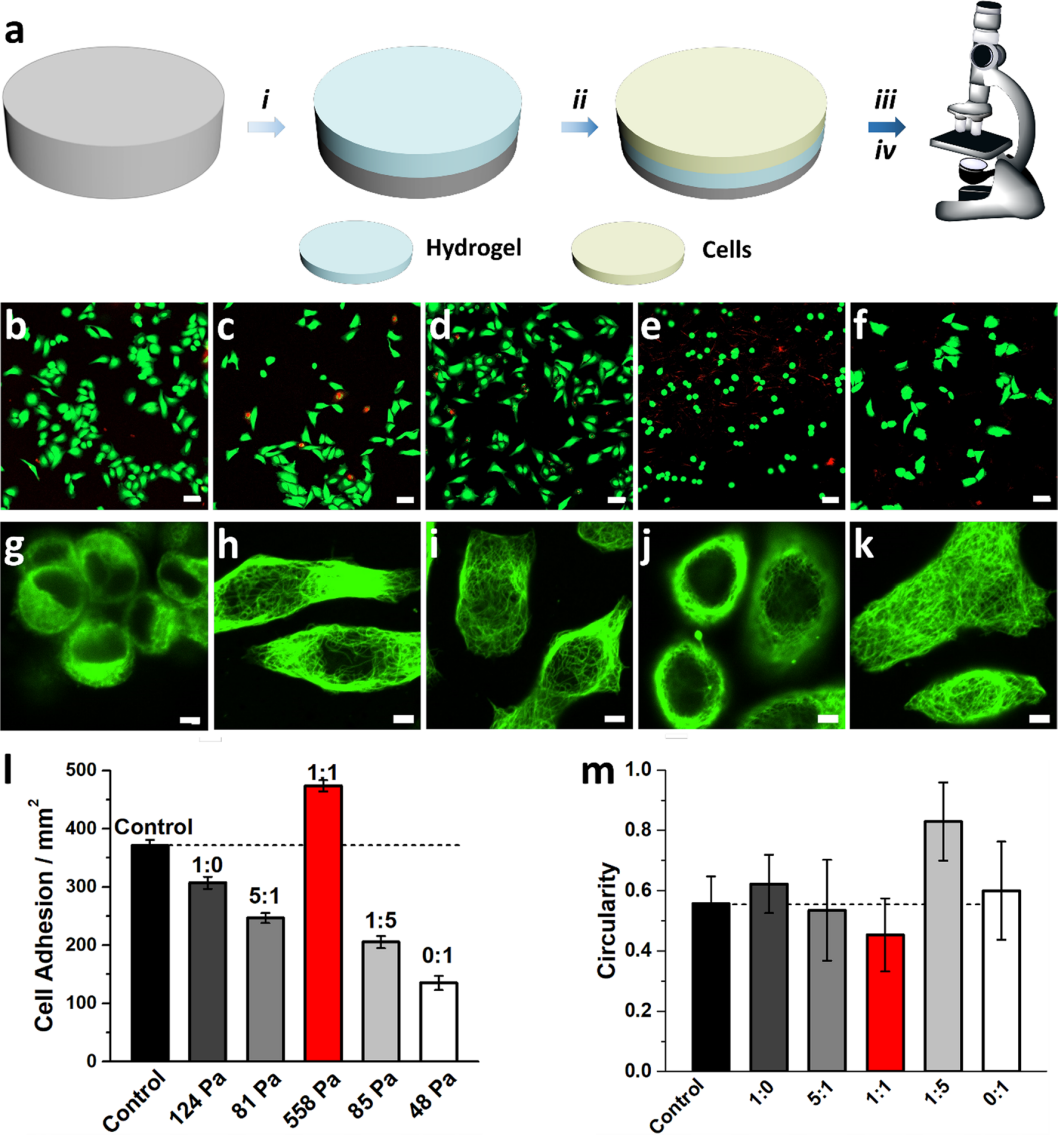
**a** 2D cell culture procedure. i. The various hydrogels are spread on the bottom of 96-well plate; ii. The different kinds of cells are seeded on the surface of hydrogels; iii. Live/dead assay or iv. Tubulin tracker staining. Live-dead assay (**b**–**f**) and tubulin staining (**g**–**k**) of HeLa cells incubated on the hydrogels formed by LMW-L1 and LMW-D1 at a molar ratio of **b**, **g** 1:0, **c**, **h** 5:1, **d**, **i** 1:1, **e**, **j** 1:5, and **f**, **k** 0:1 after 24 h. Scale bar of **b**–**f** and **g**–**k** is 50 μm and 5 μm, respectively. **l** The statistical analysis of cell adhesive and (**m**) circularity quantification of HeLa cells incubated on hydrogels formed by LMW-L1 and LMW-D1 at different molar ratio

The viscoelasticity and supramolecular chirality of a hydrogel play important roles in controlling cell adhesion and morphologies. Therefore, we investigate the cytocompatibility and cell adhesion of the above hydrogels to evaluate their potential applications in tissue engineering and regenerative medicine. Live/dead assay shows excellent biocompatibility of hydrogels in 2D cell culture (Fig. 7), as evidenced by incubating human cervical cancer cells (HeLa) on top of the 0.3 wt% hydrogels. Interestingly, the population of HeLa cells differs in the hydrogels with different mechanical properties. Specifically, more cells can be found on the hydrogel with the highest mechanical strength (Figs. 7b–f, 3j; Additional file 1: S35). We also find that HeLa cells growing on LWM-L1 hydrogel tend to form aggregate while spreading well on the hydrogel with high LMW-D1 content (Fig. 7g–k). When the HeLa cells are located at the LWM-L1 hydrogel, the cytoskeleton of cells is round, and the cells are close to each other. For the cells growing on other kinds of hydrogels, the cells are dispersive, and the microtubules extend through the whole cell body. To quantify the effect of different hydrogels on HeLa cell adhesion, we evaluate the morphology (circularity) of cells. Quantitative measurement of the adherent HeLa cells shows that the circularity index is lowest for hydrogels formed by equimolar LMW-L1 and LMW-D1 (Fig. 7i, m), which is lower than the HeLa cells incubated with culture medium, the results are consistent with the cell adherent number, indicating the favorable adhesion property [48]. To further demonstrate the biocompatibility of our hydrogels, we next perform 3D cell culture by the hydrogels since the rapid formation of hydrogels guarantees the homogeneous distribution of cells (Additional file 1: Fig. S36). Live-dead assays by CLSM show that the cells distribute homogeneously in the hydrogel. During the culture period, the cells are alive in all the tested hydrogels, as evidenced by the green fluorescence from the cells, indicating the promising application of our hydrogels in 3D cell culture. We also investigate the influence of our hydrogels on three other cell lines, including two cancer cell lines (Saos-2 and Neuro-2a) and one normal cell line (HS-5) (Additional file 1: Figs. S37–S39). The results show that the cells located on the hydrogels formed by LMW-L1 and LMW-D1 at different molar ratio represent versatile morphologies and the chiral supramolecular hydrogels formed by equimolar of LMW-L1 and LMW-D1 exhibit superior capability towards 2D cell culture, which suggest the broad feasibility of our hydrogel for controlling the adhesion and morphologies of various cell lines. These results together suggest that a simple mixture of enantiomers could be a facile strategy to tune both mechanical properties and supramolecular chirality of peptidic hydrogel for cell adhesion and morphologies.

## Conclusion

In summary, this work describes a novel approach to control the supramolecular chirality and stiffness of molecular hydrogel. Our results highlight the importance of dimerization of aromatic molecules in supramolecular chemistry and represent a general way to create and control supramolecular chirality for various applications in different disciplines [49]. For example, incorporating the ligands of two interacting proteins into our system would enable easy manipulation of their interactions in a spatiotemporal manner. The strategy demonstrated here may be useful for developing adaptable materials with controllable chirality for tissue engineering and generating biomolecular condensates in the living system.

## Supplementary Information

**Additional file 1:** All data generated or analyzed during this study are included in this published article and its supplementary information files.

## Data Availability

All the original data are available upon reasonable request for correspondence authors.

## References

[1] Wang AH-J, Quigley GJ, Kolpak FJ, Crawford JL, Van Boom JH, Van derMarel G, Rich A (1979). Molecular structure of a left-handed double helical DNA fragment at atomic resolution. Nature.

[2] Rich A, Crick FH (1955). The structure of collagen. Nature.

[3] Brown RA, Diemer V, Webb SJ, Clayden J (2013). End-to-end conformational communication through a synthetic purinergic receptor by ligand-induced helicity switching. Nat Chem.

[4] Wu H, Fuxreiter M (2016). The structure and dynamics of higher-order assemblies: amyloids, signalosomes, and granules. Cell.

[5] Liu M, Zhang L, Wang T (2015). Supramolecular chirality in self-assembled systems. Chem Rev.

[6] Wang Y, Qi W, Huang R, Yang X, Wang M, Su R, He Z (2015). Rational design of chiral nanostructures from self-assembly of a ferrocene-modified dipeptide. J Am Chem Soc.

[7] Dou X, Mehwish N, Zhao C, Liu J, Xing C, Feng C (2020). Supramolecular hydrogels with tunable chirality for promising biomedical applications. Acc Chem Res.

[8] de Jong SJ, De Smedt SC, Wahls MWC, Demeester J, Kettenes-van den Bosch JJ, Hennink WE (2000). Novel self-assembled hydrogels by stereocomplex formation in aqueous solution of enantiomeric lactic acid oligomers grafted to dextran. Macromolecules.

[9] Jiang J, Meng Y, Zhang L, Liu M (2016). Self-assembled single-walled metal-helical nanotube (M-HN): creation of efficient supramolecular catalysts for asymmetric reaction. J Am Chem Soc.

[10] Chen Xi, Huang Z, Chen S-Y, Li K, Xiao-Qi Yu, Pu L (2010). Enantioselective gel collapsing: a new means of visual chiral sensing. J Am Chem Soc.

[11] Liu GF, Zhang D, Feng CL (2014). Control of three-dimensional cell adhesion by the chirality of nanofibers in hydrogels. Angew Chem Int Ed Engl.

[12] De Greef TFA, Smulders MMJ, Wolffs M, Schenning APHJ, Sijbesma RP, Meijer EW (2009). Supramolecular polymerization. Chem Rev.

[13] Xing P, Zhao Y (2018). Controlling supramolecular chirality in multicomponent self-assembled systems. Acc Chem Res.

[14] Sun Y, Li S, Zhou Z, Saha ML, Datta S, Zhang M, Yan X, Tian D, Wang H, Wang L (2018). Alanine-based chiral metallogels via supramolecular coordination complex platforms: metallogelation induced chirality transfer. J Am Chem Soc.

[15] Wang F, Feng CL (2018). Metal-ion-mediated supramolecular chirality of l-phenylalanine based hydrogels. Angew Chem Int Ed Engl.

[16] Liu G, Sheng J, Wu H, Yang C, Yang G, Li Y, Ganguly R, Zhu L, Zhao Y (2018). Controlling supramolecular chirality of two-component hydrogels by J-and H-aggregation of building blocks. J Am Chem Soc.

[17] Tian Y, Wang H, Liu Y, Mao L, Chen W, Zhu Z, Liu W, Zheng W, Zhao Y, Kong D (2014). A peptide-based nanofibrous hydrogel as a promising DNA nanovector for optimizing the efficacy of HIV vaccine. Nano Lett.

[18] Wang M, Zhou P, Wang J, Zhao Y, Ma H, Lu JR, Xu H (2017). Left or right: how does amino acid chirality affect the handedness of nanostructures self-assembled from short amphiphilic peptides?. J Am Chem Soc.

[19] Hu Y, Lin R, Zhang P, Fern J, Cheetham AG, Patel K, Schulman R, Kan C, Cui H (2016). Electrostatic-driven lamination and untwisting of β-sheet assemblies. ACS Nano.

[20] Morris KL, Chen L, Rodger A, Adams DJ, Serpell LC (2015). Structural determinants in a library of low molecular weight gelators. Soft Matter.

[21] Cringoli MC, Romano C, Parisi E, Waddington LJ, Melchionna M, Semeraro S, De Zorzi R, Gronholm M, Marchesan S (2020). Bioadhesive supramolecular hydrogel from unprotected, short d, l-peptides with Phe–Phe and Leu-Asp-Val motifs. Chem Commun.

[22] McAulay K, Dietrich B, Su H, Scott MT, Rogers S, Al-Hilaly YK, Cui H, Serpell LC, Seddon AM, Draper ER, Adams DJ (2019). Using chirality to influence supramolecular gelation. Chem Sci.

[23] Bellotto O, Kralj S, De Zorzi R, Geremia S, Marchesan S (2020). Supramolecular hydrogels from unprotected dipeptides: a comparative study on stereoisomers and structural isomers. Soft Matter.

[24] Shy AN, Li J, Shi J, Zhou N, Xu B (2020). Enzyme-instructed self-assembly of the stereoisomers of pentapeptides to form biocompatible supramolecular hydrogels. J Drug Target.

[25] Cui H, Cheetham AG, Pashuck ET, Stupp SI (2014). Amino acid sequence in constitutionally isomeric tetrapeptide amphiphiles dictates architecture of one-dimensional nanostructures. J Am Chem Soc.

[26] Zhang SM, Greenfield MA, Mata A, Palmer LC, Bitton R, Mantei JR, Aparicio C, de la Cruz MO, Stupp SI (2010). A self-assembly pathway to aligned monodomain gels. Nat Mater.

[27] Karanicolas J, Com JE, Chen I, Joachimiak LA, Dym O, Peck SH, Albeck S, Unger T, Hu WX, Liu GH (2011). A de novo protein binding pair by computational design and directed evolution. Mol Cell.

[28] Yang X, Lu H, Tao Y, Zhou L, Wang H (2021). Spatiotemporal control over chemical assembly in living cells by integration of acid-catalyzed hydrolysis and enzymatic reactions. Angew Chem Int Ed Engl.

[29] Hu L, Li Y, Lin X, Huo Y, Zhang H, Wang H (2021). Structure-based programming of supramolecular assemblies in living cells for selective cancer cell inhibition. Angew Chem Int Ed Engl.

[30] Ma ML, Kuang Y, Gao Y, Zhang Y, Gao P, Xu B (2010). Aromatic–aromatic interactions induce the self-assembly of pentapeptidic derivatives in water to form nanofibers and supramolecular hydrogels. J Am Chem Soc.

[31] Adler-Abramovich L, Vaks L, Carny O, Trudler D, Magno A, Caflisch A, Frenkel D, Gazit E (2012). Phenylalanine assembly into toxic fibrils suggests amyloid etiology in phenylketonuria. Nat Chem Biol.

[32] Lampel A, McPhee SA, Park HA, Scott GG, Humagain S, Hekstra DR, Yoo B, Frederix P, Li TD, Abzalimov RR (2017). Polymeric peptide pigments with sequence-encoded properties. Science.

[33] Swanekamp RJ, DiMaio JT, Bowerman CJ, Nilsson BL (2012). Coassembly of enantiomeric amphipathic peptides into amyloid-inspired rippled β-sheet fibrils. J Am Chem Soc.

[34] Bera S, Xue B, Rehak P, Jacoby G, Ji W, Shimon LJW, Beck R, Kral P, Cao Y, Gazit E (2020). Self-assembly of aromatic amino acid enantiomers into supramolecular materials of high rigidity. ACS Nano.

[35] Nagy KJ, Giano MC, Jin A, Pochan DJ, Schneider JP (2011). Enhanced mechanical rigidity of hydrogels formed from enantiomeric peptide assemblies. J Am Chem Soc.

[36] Chen L, Morris K, Laybourn A, Elias D, Hicks MR, Rodger A, Serpell L, Adams DJ (2010). Self-assembly mechanism for a naphthalene-dipeptide leading to hydrogelation. Langmuir.

[37] Adhikari B, Nanda J, Banerjee A (2011). Multicomponent hydrogels from enantiomeric amino acid derivatives: helical nanofibers, handedness and self-sorting. Soft Matter.

[38] Mears LLE, Draper ER, Castilla AM, Su H, Zhuola DB, Nolan MC, Smith GN, Doutch J, Rogers S (2017). Drying affects the fiber network in low molecular weight hydrogels. Biomacromol.

[39] Dutta AK, Ray K, Mandal TK, Haque ME, Misra TN (1995). A fluorescence study of naphthalene tagged polyvinylalcohol assembled in Langmuir-Blodgett films mixed with stearic acid. Opt Mater.

[40] Martel A, Burghammer M, Davies RJ, Di Cola E, Vendrely C, Riekel C (2008). Silk fiber assembly studied by synchrotron radiation SAXS/WAXS and Raman spectroscopy. J Am Chem Soc.

[41] Escuder B, Lusar ML, Miravet JF (2006). Insight on the NMR study of supramolecular gels and its application to monitor molecular recognition on self-assembled fibers. J Org Chem.

[42] Chen Z, Stepanenko V, Dehm V, Prins P, Siebbeles LD, Seibt J, Marquetand P, Engel V, Wurthner F (2007). Photoluminescence and conductivity of self-assembled pi–pi stacks of perylene bisimide dyes. Chem Eur J.

[43] Abraham MJ, Murtola T, Schulz R, Páll S, Smith JC, Hess B, Lindahl E (2015). GROMACS: high performance molecular simulations through multi-level parallelism from laptops to supercomputers. SoftwareX.

[44] Lindorff-Larsen K, Piana S, Palmo K, Maragakis P, Klepeis JL, Dror RO, Shaw DE (2010). Improved side-chain torsion potentials for the Amber ff99SB protein force field. Proteins.

[45] Jorgensen WL, Chandrasekhar J, Madura JD, Impey RW, Klein ML (1983). Comparison of simple potential functions for simulating liquid water. J Chem Phys.

[46] Essmann U, Perera L, Berkowitz ML, Darden T, Lee H, Pedersen LG (1995). A smooth particle mesh Ewald method. J Chem Phys.

[47] WilliamHumphrey AD, Schulten K (1996). VMD: visual molecular dynamics. J Mol Graphics.

[48] Gribova V, Gauthier-Rouviere C, Albiges-Rizo C, Auzely-Velty R, Picart C (2013). Effect of RGD functionalization and stiffness modulation of polyelectrolyte multilayer films on muscle cell differentiation. Acta Biomater.

[49] Jeena M, Jeong K, Go EM, Cho Y, Lee S, Jin S, Hwang S-W, Jang JH, Kang CS, Bang W-Y (2019). Heterochiral assembly of amphiphilic peptides inside the mitochondria for supramolecular cancer therapeutics. ACS Nano.

