# Supplementary material for: Controlling supramolecular filament chirality of hydrogel by co-assembly of enantiomeric aromatic peptides
Source: J Nanobiotechnology. 2022 Feb 10;20:77. doi: 10.1186/s12951-022-01285-0 (PMC8832752; doi:10.1186/s12951-022-01285-0)
Supplement: Supplementary file 1 — Additional file 1: All data generated or analyzed during this study are included in this published article and its supplementary information files. [file 12951_2022_1285_MOESM1_ESM.docx]

Supporting Information for

Controlling Supramolecular Filament Chirality of Hydrogel by Co-assembly of Enantiomeric Aromatic Peptides

Xuejiao Yang,^1^ Honglei Lu,^1^ Yinghua Tao,^1^ Hongyue Zhang^1^ and Huaimin Wang^1, 2^

^1^Key Laboratory of Precise Synthesis of Functional Molecules of Zhejiang Province, School of Science, Westlake University; Westlake Institute for Advanced Study, 18 Shilongshan Road, Hangzhou 310024, Zhejiang Province, China
^2^Westlake Laboratory of Life Sciences and Biomedicine, School of Life Sciences, Westlake University, Hangzhou, Zhejiang, China.

Corresponding author: Huaimin Wang

E-mail address: wanghuaimin@westlake.edu.cn

**Content**

**S1. Experiment materials and instruments**…...…………………….............................................5

**S2. Experiment methods**…...……………………...........................................................................6

**Scheme S1.** Synthetic route of **LMW-L1**, **LMW-D1**, **LMW-L2**, **LMW-D2**, **LMW-L3**, and **LMW-D3**.

**S3. Characterizations**..………………………...………..................................................…............9

**S4. Supporting figures**…………………………….…....................................……………...........12

**Fig. S1.** To test mechanical strength, we placed peptide solution at the ringed component, which was obtained from a syringe.

**Fig. S2.** ^1^H NMR spectrum of **LMW-L1**.

**Fig. S3.** ^1^H NMR spectrum of **LMW-D1**.

**Fig. S4.** ^1^H NMR spectrum of **LMW-L2**.

**Fig. S5.** ^1^H NMR spectrum of **LMW-D2**.

**Fig. S6.** ^1^H NMR spectrum of **LMW-L3**.

**Fig. S7.** ^1^H NMR spectrum of **LMW-D3**.

**Fig. S8.** LC-MS spectrum of **LMW-L1**.

**Fig. S9.** LC-MS spectrum of **LMW-D1**.

**Fig. S10.** LC-MS spectrum of **LMW-L2**.

**Fig. S11.** LC-MS spectrum of **LMW-D2**.

**Fig. S12.** LC-MS spectrum of **LMW-L3**.

**Fig. S13.** LC-MS spectrum of **LMW-D3**.

**Fig. S14.** Optical images of hydrogels formed by **LMW-L1** and **LMW-D1** at different molar ratio. From left to right, the molar ratio is 1:0, 10:1, 5:1, 2:1, 1:1, 1:2, 1:5, 1:10, and 0:1, respectively. The total concentration of **LMW-L1** and **LMW-D1** is 0.3 wt%.

**Fig. S15.** AFM images and corresponding amplitude images of nanofibers formed by **LMW-L1** and **LMW-D1** at a molar ratio of 1:0, 0:1, and 1:1, respectively. The total concentration of **LMW-L1** and **LMW-D1** is 0.3 wt%.

**Fig. S16.** a-c) SEM and d-i) high magnification TEM images of nanofibers formed by L**MW-L1** and **LMW-D1** at a molar ratio of a) 1:0, b) 0:1, and c) 1:1, respectively. The total concentration of **LMW-L1** and **LMW-D1** is 0.3 wt%.

**Fig. S17.** Statistic diameter of nanofibers formed by **LMW-L1** and **LMW-D1** at a molar ratio of a) 1:0, b) 0:1, and c) 1:1, respectively. The total concentration of **LMW-L1** and **LMW-D1** is 0.3 wt%.

**Fig. S18.** TEM images of hydrogels formed by equimolar mixture of **LMW-L1** and **LMW-D1**. The total concentration of **LMW-L1** and **LMW-D1** is 0.3 wt%. Scale bar is 100 nm.

**Fig. S19.** HT data of hydrogels formed by **LMW-L1** and **LMW-D1** at a molar ratio of a) 1:0, b) 10:1, c) 5:1, d) 2:1, e) 1:1, f) 1:2, g) 1:5, h) 1:10, and i) 0:1. The HT trace is equivalent to the hydrogel’s absorbance. The total concentration of LMW-L1 and LMW-D1 is 0.3 wt%.

**Fig. S20.** TEM images of hydrogels formed by **LMW-L1** and **LMW-D1** at a molar ratio of a) 1:0, b) 10:1, c) 5:1, d) 2:1, e) 1:1, f) 1:2, g) 1:5, h) 1:10, and i) 0:1. Scale bar is 200 nm. The total concentration of **LMW-L1** and **LMW-D1** is 0.3 wt%.

**Fig. S21.** Strain sweep (0.01% -100%) of hydrogels formed by **LMW-L1** and **LMW-D1** at a molar ratio of a) 1:0, b) 10:1, c) 5:1, d) 2:1, e) 1:1, f) 1:2, g) 1:5, h) 1:10, and i) 0:1, respectively. The total concentration of **LMW-L1** and **LMW-D1** is 0.3 wt%.

**Fig. S22.** Three individual results of frequency sweep (0.1-100 Hz) of hydrogels that formed by **LMW-L1** and **LMW-D1** at a molar ratio of a) 1:0, b) 10:1, c) 5:1, d) 2:1, e) 1:1, f) 1:2, g) 1:5, h) 1:10, and i) 0:1, respectively. The fixed strain is 0.5%. The total concentration of **LMW-L1** and **LMW-D1** is 0.3 wt%.

**Fig. S23.** a) UV-vis spectra, b) the wavelength offset in UV-vis spectra, c) fluorescence spectra, and d) CD spectra, of hydrogels formed by **LMW-L1** and **LMW-D1** at different molar ratio. The total concentration of **LMW-L1** and **LMW-D1** is 0.3 wt%.

**Fig. S24.** *In situ* WAXS patterns of hydrogels formed by **LMW-L1** and **LMW-D1** with a molar ratio of a) 10:1, b) 5:1, c) 2:1, d) 1:2, e) 1:5, and f) 1:10, respectively. The total concentration of **LMW-L1** and **LMW-D1** is 0.3 wt%.

**Fig. S25.** ^1^H NMR spectrum of nanostructures formed by **LMW-L1** and **LMW-D1** in deuterated PBS at different molar ratio. The total concentration of **LMW-L1** and **LMW-D1** is 0.1 wt%.

**Fig. S26.** Molecular structure of a) **LMW-2**, b) **LMW-3**, c, d) optical images and e, f) CD spectra of nanostructures formed by a, c) **LMW-L2** and **LMW-D2**, b, d) **LMW-L3** and **LMW-D3**. In the optical photographs, from left to right, the molar ratio is 1:0, 0:1 and 1:1, respectively. The total concentration of **LMW-L2** and **LMW-D2**, **LMW-L3** and **LMW-D3** is 0.3 wt%.

**Fig. S27.** TEM images of nanostructures formed by a-c) **LMW-L2** and **LMW-D2**, d-f) **LMW-L3** and **LMW-D3**, from left to right, the molar ratio is 1:0, 0:1 and 1:1, respectively. The scale bar for a-c) and d-f) is 50 nm and 200 nm, respectively. The total concentration of **LMW-L2** and **LMW-D2**, **LMW-L3** and **LMW-D3** is 0.3 wt%.

**Fig. S28.** 2D DOSY spectrum of nanostructures formed by **LMW-L1** in deuterated PBS at a concentration of 0.1 wt%. The lg D was measured as -9.655 m^2^ s^-1^.

**Fig. S29.** 2D DOSY spectrum of nanostructures formed by **LMW-D1** in deuterated PBS at a concentration of 0.1 wt%. The lg D was measured as -9.597 m^2^ s^-1^.

**Fig. S30.** 2D DOSY spectrum of nanostructures formed by **LMW-L1** and **LMW-D1** in deuterated PBS at a concentration of 0.1 wt%. The lg D was measured as -9.643 m^2^ s^-1^.

**Fig. S31.** 2D NOESY spectrum of nanostructures formed by **LMW-L1** in deuterated PBS at a concentration of 0.1 wt%.

**Fig. S32.** 2D NOESY spectrum of nanostructures formed by **LMW-D1** in deuterated PBS at a concentration of 0.1 wt%.

**Fig. S33.** 2D NOESY spectrum of nanostructures formed by equal molar of **LMW-L1** and **LMW-D1** in deuterated PBS at a total concentration of 0.1 wt%. Close contacts are showed with colored circles.

**Fig. S34.** Optimized structures of aggregates formed by **LMW-L1** and **LMW-D1** at a molar ratio of a) 1:0, b) 0:1, and c) 1:1 *via* MD calculations, respectively. The total concentration of **LMW-L1** and **LMW-D1** is 0.3 wt%.

**Fig. S35.** Morphologies of HeLa cells incubated with a) culture medium and hydrogels formed by **LMW-L1** and **LMW-D1** at a molar ratio of b) 1:0, c) 5:1, d) 1:1, e) 1:5, and f) 0:1 after 24 h of live/dead test.

**Fig. S36.** 3D cell culture of HeLa cells in the hydrogels co-assembled by **LMW-L1** and **LMW-D1** at a molar ratio of a) 1:0, b) 1:1, and c) 0:1.

**Figure S37.** Morphologies of Saos-2 cells incubated with hydrogels formed by **LMW-L1** and **LMW-D1** at a molar ratio of a) 1:0, b) 1:1, and c) 0:1 after 24 h of live/dead test. d) The statistic adhesive Saos-2 cells on hydrogels formed by **LMW-L1** and **LMW-D1** at different molar ratios.

**Figure S38.** Morphologies of Neuro-2a cells incubated with hydrogels formed by **LMW-L1** and LMW-D1 at a molar ratio of a) 1:0, b) 1:1, and c) 0:1 after 24 h of live/dead test. d) The statistic adhesive Neuro-2a cells on hydrogels formed by **LMW-L1** and **LMW-D1** at different molar ratios.

**Figure S39.** Morphologies of HS-5 cells incubated with hydrogels formed by **LMW-L1** and **LMW-D1** at a molar ratio of a) 1:0, b) 1:1, and c) 0:1 after 24 h of live/dead test. d) The statistic adhesive HS-5 cells on hydrogels formed by **LMW-L1** and **LMW-D1** at different molar ratios.

**S5. Supporting tables**………………………........................................………........……..............33

**Table S1.** LCM-MS method.

**Table S2.** The relationship between gelation time and storage modulus of different hydrogels co-assembled by **LMW-L1** and **LMW-D1**.

**Table S3.** Storage modulus and loss modulus in stress-strain and frequency sweep experiment of hydrogels formed by **LMW-L1** and **LMW-D1** with various molar ratio. The fixed strain is 0.5%. The total concentration of **LMW-L1** and **LMW-D1** is 0.3 wt%.

**S1. Experiment materials and instruments**

All chemicals were purchased from commercial sources and used without further purification. 2-Cl-trityl chloride resin (1.04 mmol/g) and Fmoc-amino acids were obtained from GL Biochem (Shanghai, China). Other chemical reagents and solvents were obtained from Aladdin Industrial Co., Ltd (Shanghai, China). Minimal essential medium (MEM), fetal bovine serum (FBS), live cell imaging solution and live/dead assay were purchased from Gibco (thermal fisher scientific, US).

The nuclear magnetic resonance spectroscopy (NMR) experiments were recorded on AVANCE NEO spectrometer (Bruker, US). The liquid chromatography-mass spectrometer (LC-MS) experiments were detected using LC-MS (Agilent 1260 Infinity) equipped with C18 column. The cryo-electron microscopy (Cryo-EM) was carried on 200 kV Cryo-EM (Glacios, US). The transmission electron microscope (TEM) was performed on Talos L120C TEM (Thermo Fisher, US). The circular dichroism (CD) experiments were recorded by circular dichroism (Applied Photophysics Ltd, UK). The fourier transform infrared spectroscopy (FTIR) was investigated on a Nicolet iS50 FTIR spectrometer (Thermo Fisher, US) by ATR method. The ultraviolet-visible spectrum (UV-vis) and fluorescence spectra was carried on Varioskan lux microplate reader (Thermo Fisher, US). The rheological measurement was carried out using an ARES-G2 Rheometer (TA Waters, US). The *in situ* wide angle X-ray scattering (*in situ* WAXS) spectrum was investigated using a D8 Venture DUO microsourced single crystal X-ray diffractometer (Bruker, Germany) with an Ius Diamond Cu. The laser scanning confocal microscopy (CLSM) was performed on Zeiss LSM800 CLSM (Zeiss, Germany).

**S2. Experiment methods**

**2.1 Synthesis and purification of LMW-1, LMW-2, and LMW-3:**

We synthesized **LMW-1**, **LMW-2**, **and LMW-3** by standard solid phase peptide synthesis (SPPS). Scheme S1 shows the general synthetic route. All the peptides were purified by reverse phase HPLC equipped with C18 column using HPLC grade acetonitrile and deionized water with supplement of 0.1% trifluoroacetic acid as the eluents. The purified peptides were detected by ^1^H NMR and LC-MS.

**Scheme S1.** Synthetic route of **LMW-L1**, **LMW-D1**, **LMW-L2**, **LMW-D2**, **LMW-L3**, and **LMW-D3**. i. Fmoc-_L/D_-Phe-OH, HBTU, DIPEA, 1 h; ii) 20% piperidine, 30 min; iii) Fmoc-_L/D_-Tyr(tBu)-OH, HBTU, DIPEA, 2 h; iv) Fmoc-_L/D_-Trp(Boc)-OH, HBTU, DIPEA, 2 h; a-v) naphthylacetic acid, HBTU, DIPEA, 2 h; a-vi) 95% TFA, 2.5% H_2_O, 2.5% TIS; b-v) acetic anhydride, HBTU, DIPEA, 2 h; b-vi) 95% TFA, 2.5% H_2_O, 2.5% TIS; c-v) 1-pyrenebutyric acid, HBTU, DIPEA, 2 h; c-vi) 95% TFA, 2.5% H_2_O, 2.5% TIS.

**2.2 Preparation of hydrogels formed by LMW-L1:**

We prepared hydrogel by heating-cooling strategy. Briefly, we dissolved **LMW-L1** in phosphate buffer (PBS) at 75 ºC for 1 minutes, then the sample was cooled and incubated at room temperature without any disturbance. After 10 minutes, we could see the formation of stable hydrogels. The critical gelation concentration (CGC) of **LMW-L1** is 0.2 wt%.

**2.3 Preparation of hydrogels formed by LMW-D1:**

We prepared hydrogel formed by **LMW-D1** with the same method as **LMW-L1** described in above procedure. The CGC of **LMW-D1** is 0.2 wt%.

**2.4 Preparation of hydrogels formed by LMW-L1 and LMW-D1:**

We prepared enantiomeric hydrogels by heating-cooling strategy. Briefly, we dissolved **LMW-L1** and **LMW-D1** at different molar ratio (10:1, 5:1, 2:1, 1:1, 1:2, 1:5, 1:10) in phosphate buffer (PBS) at 75 ºC for 1 minutes, then the samples were cooled and incubated at room temperature without any disturbance. Because of the decrease of solubility induced by cooling, the previous dissolved peptide monomers could aggregate into the ordered self-assembled structure, which further entangled with each other to form a hydrogel. After 5-10 minutes, we can see the formation of hydrogels. The total concentration of **LMW-L1** and **LMW-D1** is 0.3 wt%. The gelation time of different hydrogels is shown in Table S1.

**2.5 Preparation of nanostructures formed by LMW-L2 and LMW-D2:**

We dissolved **LMW-L2** and **LMW-D2** at different molar ratio (1:0, 1:1, 0:1) in phosphate buffer (PBS) at room temperature, then the samples were incubated without any disturbance. For the three kinds of samples, we didn’t observe the formation of hydrogels. The total concentration of **LMW-L2** and **LMW-D2** is 0.3 wt%.

**2.6 Preparation of nanostructures formed by LMW-L3 and LMW-D3:**

We prepared different kinds of nanostructures by heating-cooling strategy. We dissolved **LMW-L3** and **LMW-D3** at different molar ratio (1:0, 1:1, 0:1) in phosphate buffer (PBS) at 80 ºC for 1 minutes because of the poor solubility, then the samples were cooled and incubated at room temperature without any disturbance. For the mixture of equal molar of **LMW-L3** and **LMW-D3**, we could observe the formation of hydrogels after 1 h. The total concentration of **LMW-L3** and **LMW-D3** is 0.3 wt%.

**2.7 2D cell culture:**

First, the powder of **LMW-L1** and **LMW-D1** at various molar ratio (1:0, 5:1, 1:1, 1:5, 0:1) were dispersed at PBS (0.3 wt%, pH=7.4), then the mixture was heated for 15 s to form homogenous solution, after placing 50 μL peptide solution into 96-well plate, the solution was spread on the bottom uniformly. After incubating for 2 h, the hydrogels formed stably, then we added 50 μL fresh culture medium to the top of the above hydrogels for 30 min. After buffer changing for 3 times, the PBS inside the hydrogels could be replaced by culture medium completely. After incubating the hydrogels for another 24 h, we seeded the HeLa cells, Saos-2 cells, Neuro-2a cells, and HS-5 cells (1*10^4^ cells/well) on the resulting 0.3 wt% hydrogel and incubated at CO_2_ incubator (37 °C) for 24 h. We stained the cells using live/dead assay and tubulin tracker, then used CLSM to investigate the cell morphology and adhesion property that incubated on different hydrogels.

**2.8 3D cell culture:**

The 3D cell culture was achieved by encapsulating HeLa cells into various hydrogels. First, the powder of **LMW-L1** and **LMW-D1** at various molar ratio (1:0, 1:1, 0:1) was dispersed at PBS (0.3 wt%), then the mixture was heated for 15 s to result in homogenous solution, then we placed 100 μL peptide solution to 96-well plate. After incubating for 2 h, the hydrogels formed stably, then we added 100 μL fresh culture medium to incubate with hydrogels for 30 min, then we removed excess PBS. After 3 times, the PBS inside the hydrogels could be replaced by culture medium completely. We added the HeLa cells into the hydrogels, the hydrogel and the cells were mixed uniformly, the final concentration of HeLa cells was 1*10^6^ cells/ mL, and the volume ratio of added HeLa cell to hydrogels was smaller than 20%. After incubating for 24 h, we stained the cells using live/dead assay and used CLSM to investigate the morphology of cells.

**2.9 Quantification of cell adhesion:**

For the CLSM images of different kinds of cells incubated with hydrogels, we used ImageJ to quantify the cell adhesion numbers and investigate the adhesion property. Cell circularity is a parameter defined by the formula: Circularity = 4π(A/P^2^), (A is the cell area and P is the perimeter), when the circularity index is 1.0, the cell is a perfect circle; when the circularity index towards 0, indicating the elongated polygon.

1) Input the CLSM image;

2) Duplicate the image;

3) Adjust the “threshold” to make the cells are included as much as possible;

4) Click “Watershed” to make the cells separate;

5) Click “Analyze Particles”, the circularity scope is 0.00-1.00, then we obtain the cell adhesion numbers and the circularity index;

6) For the adhesion numbers and circularity index, we counted 3 individual samples (each sample has 3 CLSM images), then averaged the data and added the error bars.

**S3. Characterizations**

**S3.1 Nuclear magnetic resonance spectroscopy (NMR)**

The ^1^H NMR was recorded on 500 M AVANCE NEO spectrometer (Bruker, US), referenced to Si(CH_3_)_4_. The solvent used in the measurement was d_6_-DMSO.

For DOSY and NOESY experiments, we dissolved **LMW-L1** and **LMW-D1** at different molar ratio in deuterated phosphate buffer (PBS) at 75 ℃ for 1 minute, then the samples were cooled and incubated at room temperature without any disturbance for 24 h. The total concentration of **LMW-L1** and **LMW-D1** is 0.1 wt%. After 24 h, we first performed ^1^H NMR spectra of peptides in aggregated state, then we confirmed the chimical shift of protons according to the ^1^H NMR spectra of monomers. Then the DOSY and NOESY spectrum were measured in deuterated PBS with 256 scans on 600 M AVANCE NEO spectrometer (Bruker, US). The degree of polymerization was calculated according to Stokes-Einstein equation. According to the chemical shift of H protons in the aggregated state, we confirm the NOE interactions.

For the temperature dependent ^1^H NMR experiment, we dissolved **LMW-L1** and **LMW-D1** at a molar ratio of 1:1 in deuterated phosphate buffer (PBS) at 75 ºC for 1 minute, then the samples were cooled and incubated at room temperature without any disturbance for 24 h. The total concentration of **LMW-L1** and **LMW-D1** is 0.1 wt%. The ^1^H NMR spectrum was recorded on 500 M AVANCE NEO spectrometer (Bruker, US) at a temperature range from 25 ºC to 95 ºC.

**S3.2 Liquid chromatography-mass spectrometer (LC-MS)**

The synthesized peptides were dissolved in MeOH at a concentration of 0.2-0.5 mg mL^-1^, then the purity and molecular weight of samples were detected using LC-MS (Agilent 1260 Infinity) equipped with C18 column, the mobile phase was acetonitrile (0.5‰ TFA) and deionized water (0.5‰ TFA), the detailed method is shown in Table S1.

**S3.3 Cryo-electron microscopy (Cryo-EM)**

First, we placed 10 μL of hydrogels on the grid, then the excess samples on the grid were removed with a filter paper. Afterward, the grid was immediately plunged into precooled liquid ethane. We observed the samples immediately by 200 kV Cryo-EM (Glacios, US).

**S3.4 Transmission electron microscope (TEM)**

10 μL aliquot of hydrogel was placed onto a 200 meshes carbon-coated copper grid, then the excess samples were removed using a filter paper, then we added 10 μL of uranyl acetate (UA) to stain the sample, and the samples were air dried. The samples were investigated using Talos L120C TEM (Thermo Fisher, US) operated at 120 kV.

**S3.5 Atomic force microscope (AFM)**

100 μL aliquot of hydrogels was dispersed on a mica sheet, then the samples were dried using nitrogen purging. The morphology of the self-assemblies was recorded with an atomic force microscope (AFM, Cypher ES, US) an AIST-NT Smart AFM system in non-contact (tapping) mode.

**S3.6 Scanning electron microscope (SEM)**

100 μL aliquot of hydrogels was placed on a silicon wafer, then the samples were air dried and sputtered with gold (EMS/150TS, UK). The morphology of the self-assemblies was observed with SEM (Regulus 8230, Hitachi High-Technologies CO., Japan) with an acceleration voltage of 5 keV.

**S3.7 Circular dichroism (CD)**

100 μL aliquot of hydrogel was added into the quartz cell with a 0.1 cm path length, and the CD signal was recorded from 180 nm to 280 nm with a scan speed of 100 nm/min by circular dichroism (Applied Photophysics Ltd, UK).

**S3.8 Fourier transform infrared spectroscopy (FTIR)**

We prepared the hydrogels using deuterated PBS. First, we freeze-dried 600 uL of PBS, then we added equal volume of D_2_O into the powder, contributing to the deuterated PBS. After that, we prepared hydrogels formed by **LMW-L1** and **LMW-D1** at different molar ratio using deuterated PBS by “heating-cooling” strategy, after incubating at room temperature for 24 h, we placed the hydrogel on the CaF clips, then the FTIR spectra was recorded on a Nicolet iS50 FTIR spectrometer (Thermo Fisher, US) across the range of 1100-4000 cm^-1^, and the background of deuterated PBS was deducted.

**S3.9 Ultraviolet-visible spectrum (UV-vis) and fluorescence spectra**

We added hydrogel (100 μL) into 96-well plates, then recorded the UV-vis spectra (270-350 nm) and fluorescence spectra (350-500 nm) using Varioskan lux microplate reader (Thermo Fisher, US).

**S3.10 Rheological measurement**

Rheological properties of assembled hydrogels were carried out using an ARES-G2 Rheometer (TA Waters, US). All the experiments were performed at 25 °C. We prepared three individual experiments for each hydrogel. First, we dissolved **LMW-L1** and **LMW-D1** at different molar ratio (1:0, 10:1, 5:1, 2:1, 1:1, 1:2, 1:5, 1:10, 0:1) in phosphate buffer (PBS) at 75 ºC for 1 minutes (the total concentration of **LMW-L1** and **LMW-D1** was 0.3 wt%), then we placed 500 μL of peptide solution at the ringed component made by ourselves (Fig. S1), after incubating at 25 °C for 15 min, we removed ringed component, and the hydrogels were very stable without change. We next performed the stress-strain and frequency sweep experiments successively. Stress-strain sweeps were performed on hydrogels at the strain from 0.01 % to 100 % to determine the limit of the linear viscoelastic region. The frequency sweep experiments were recorded as a function of angular frequency (0.01-100 Hz) at the strain of 0.5%.


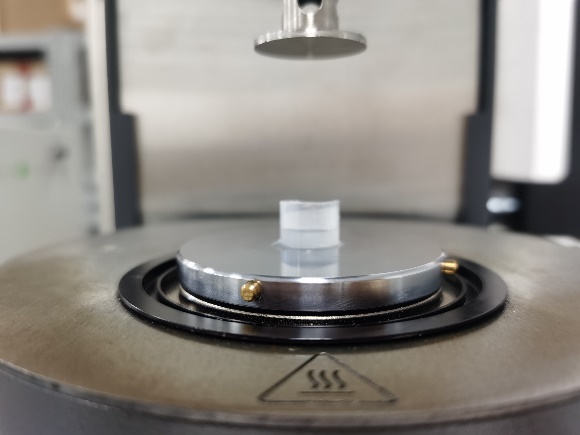


**Fig. S1.** To test mechanical strength, we placed peptide solution at the ringed component, which was obtained from a syringe.

**S3.11 *In situ* wide angle X-ray scattering (*in situ* WAXS)**

In situ WAXS spectrum was investigated using a D8 Venture DUO microsourced single crystal X-ray diffractometer (Bruker, Germany) with an Ius Diamond Cu, the prepared hydrogels were directly put in the sample cell. The distance of sample-detector is 38 mm, and the exposure time is 60 s.

**S3.12 Laser scanning confocal microscopy (CLSM)**

To visualize the HeLa cell viability on the hydrogels, the cells were washed with fresh culture medium for 3 times, and then stained with live/dead assay. After 20 min of incubation, we washed the cells by live cell imaging buffer for 3 times, and then imaged the cells with Zeiss LSM800 CLSM (Zeiss, Germany).

**S3. Supporting figures**


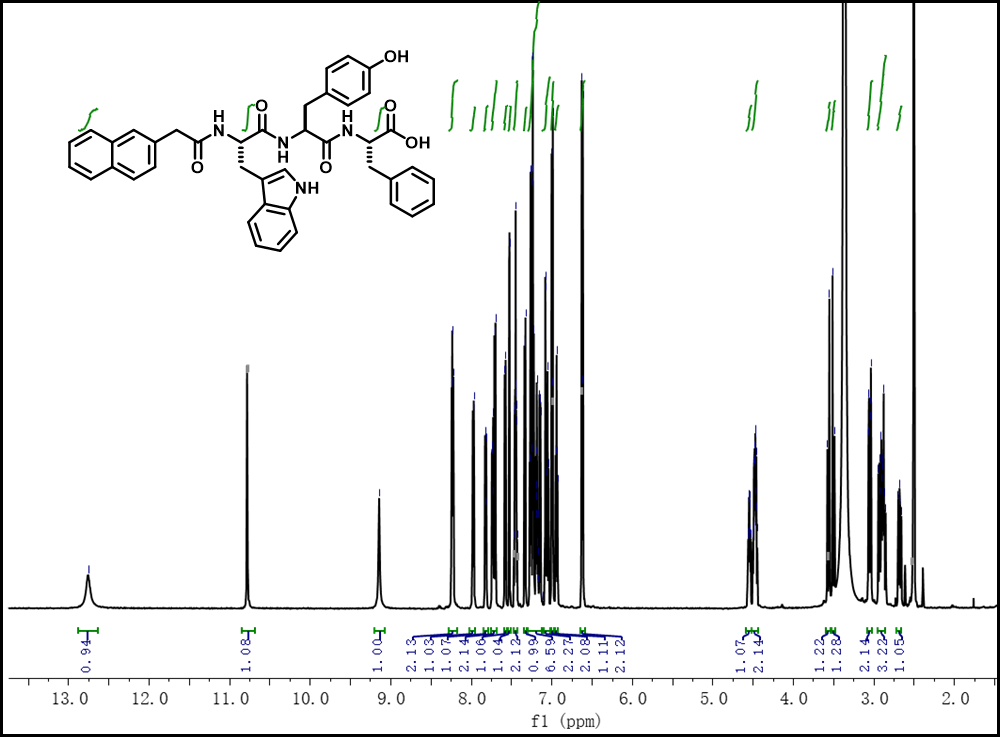


**Fig. S2.** ^1^H NMR spectrum of **LMW-L1**. δ12.76 (29, br, 1H, COOH), δ10.78 (35, s, 1H, NH in tryptophan), δ9.14 (51, s, 1H, OH), δ8.23 (14, 19, m, 2H, NH), δ7.96 (24, m, 1H, NH), δ7.81 (7, m, 1H, CH in naphthalene), δ7.71 (3, 10, m, 2H, CH in naphthalene), δ7.58 (4, s, 1H, CH in naphthalene), δ7.52 (6, m, 1H, CH in naphthalene), δ7.45 (9, 8, m, 2H, CH in naphthalene), δ7.33 (37, m, 1H, CH in tryptophan), δ7.25 (40, 36, 46, 47, m, 4H, CH in tryptophan and phenylalanine), δ7.18 (48, m, 1H, CH in phenylalanine), δ7.14 (49, m, 1H, CH in phenylalanine), δ7.05 (50, 41, m, 2H, CH in tyrosine), δ6.99 (45, 39, m, 2H, CH in tyrosine), δ6.94 (38, m, 1H, CH in tyrosine), δ6.61 (42, 44, m, 2H, α-H in tyrosine), δ4.54 (15, m, 1H, -CH), δ4.47 (20, 25, m, 2H, -CH), δ3.56 (11’, m, 1H, -CH_2_), δ3.50 (11’, m, 1H, -CH_2_), δ3.05 (26, m, 2H, -CH_2_), δ2.91 (21, 16’, m, 3H, -CH_2_), δ2.69 (16’, m, 1H, -CH_2_).


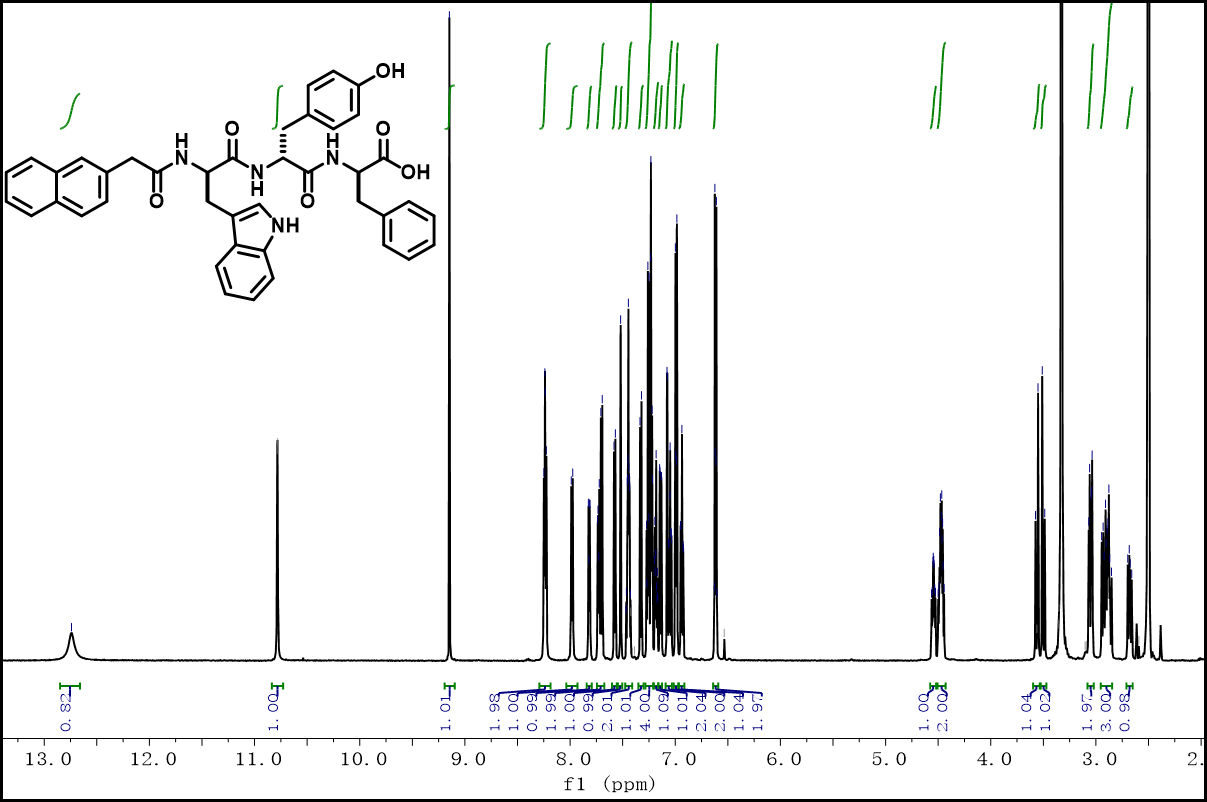


**Fig. S3.** ^1^H NMR spectrum of **LMW-D1**. δ12.74 (29, br, 1H, COOH), δ10.78 (35, s, 1H, NH in tryptophan), δ9.15 (51, s, 1H, OH), δ8.24 (14, 19, m, 2H, NH), δ7.99 (24, m, 1H, NH), δ7.82 (7, m, 1H, CH in naphthalene), δ7.72 (3, 10, m, 2H, CH in naphthalene), δ7.58 (4, s, 1H, CH in naphthalene), δ7.52 (6, m, 1H, CH in naphthalene), δ7.44 (9, 8, m, 2H, CH in naphthalene), δ7.32 (37, m, 1H, CH in tryptophan), δ7.25 (40, 36, 46, 47, m, 4H, CH in tryptophan and phenylalanine), δ7.18 (48, m, 1H, CH in phenylalanine), δ7.13 (49, m, 1H, CH in phenylalanine), δ7.06 (50, 41, m, 2H, CH in tyrosine), δ6.99 (45, 39, m, 2H, CH in tyrosine), δ6.95 (38, m, 1H, CH in tyrosine), δ6.61 (42, 44, m, 2H, α-H in tyrosine), δ4.55 (15, m, 1H, -CH), δ4.47 (20, 25, m, 2H, -CH), δ3.55 (11’, m, 1H, -CH_2_), δ3.50 (11’, m, 1H, -CH_2_), δ3.05 (26, m, 2H, -CH_2_), δ2.91 (21, 16’, m, 3H, -CH_2_), δ2.68 (16’, m, 1H, -CH_2_).


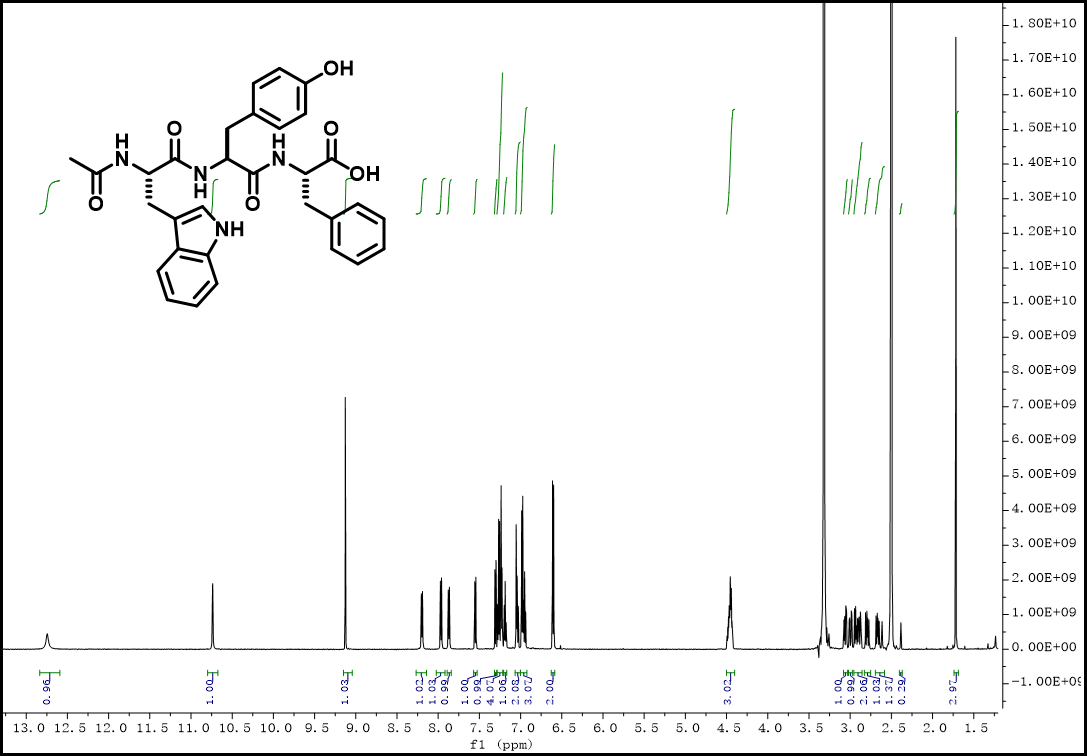


**Fig. S4.** ^1^H NMR spectrum of **LMW-L2**. δ12.74 (br, 1H, COOH), δ10.74 (s, 1H, NH in tryptophan), δ9.13 (s, 1H, OH in tyrosine), δ8.20 (m, H, NH), δ7.96 (m, 1H, NH), δ7.88 (m, 1H, NH), δ7.56 (m, 1H, CH in tryptophan), δ7.31-6.93 (m, 11H, CH in tryptophan, tyrosine, and phenylalanine), δ6.61 (m, 2H, CH in tyrosine), δ4.46 (m, 3H, CH), δ3.07-2.65 (m, 6H, -CH_2_), δ1.72 (s, 3H, -CH_3_).


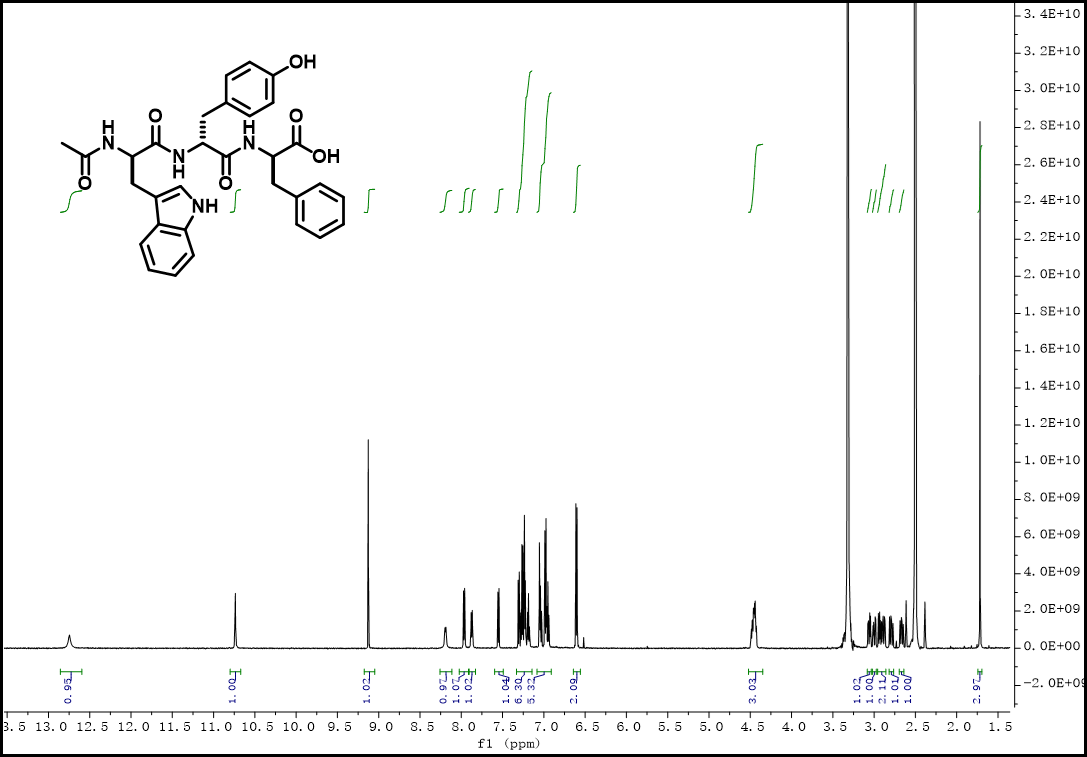


**Fig. S5.** ^1^H NMR spectrum of **LMW-D2**. δ12.75 (br, 1H, COOH), δ10.74 (s, 1H, NH in tryptophan), δ9.13 (s, 1H, OH in tyrosine), δ8.19 (m, H, NH), δ7.96 (m, 1H, NH), δ7.87 (m, 1H, NH), δ7.54 (m, 1H, CH in tryptophan), δ7.31-6.95 (m, 11H, CH in tryptophan, tyrosine, and phenylalanine), δ6.60 (m, 2H, CH in tyrosine), δ4.46 (m, 3H, CH), δ3.07-2.63 (m, 6H, -CH_2_), δ1.72 (s, 3H, -CH_3_).


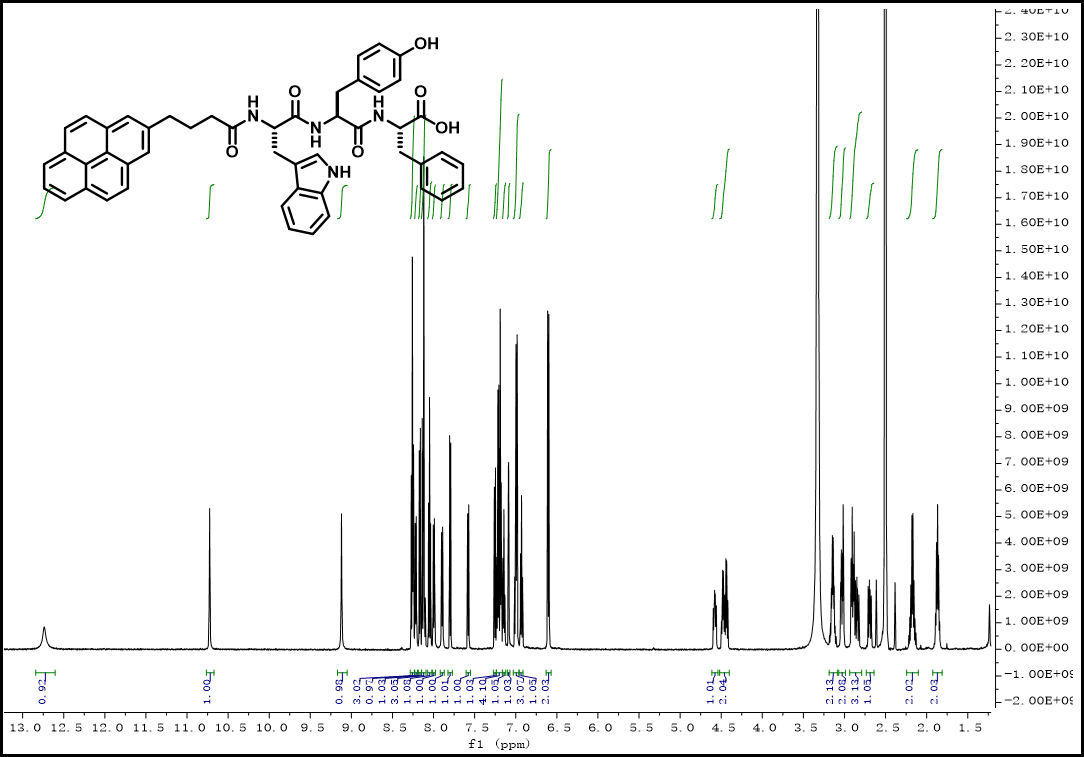


**Fig. S6.** ^1^H NMR spectrum of **LMW-L3**. δ12.75 (br, 1H, COOH), δ10.73 (s, 1H, NH in tryptophan), δ9.12 (s, 1H, OH in tyrosine), δ8.26 (m, 3H, NH), δ8.21-7.79 (m, 9H, CH in pyrene), δ7.59 (m, 1H, CH in tryptophan), δ7.26-6.92 (m, 11H, CH in tryptophan, tyrosine, and phenylalanine), δ6.60 (m, 2H, CH in tyrosine), δ4.58-4.45 (m, 3H, CH), δ3.15-2.67 (m, 8H, -CH_2_ in pyrene, tryptophan, phenylalanine, and tyrosine), δ2.19-1.85 (m, 4H, -CH_2_ in pyrene and tryptophan).


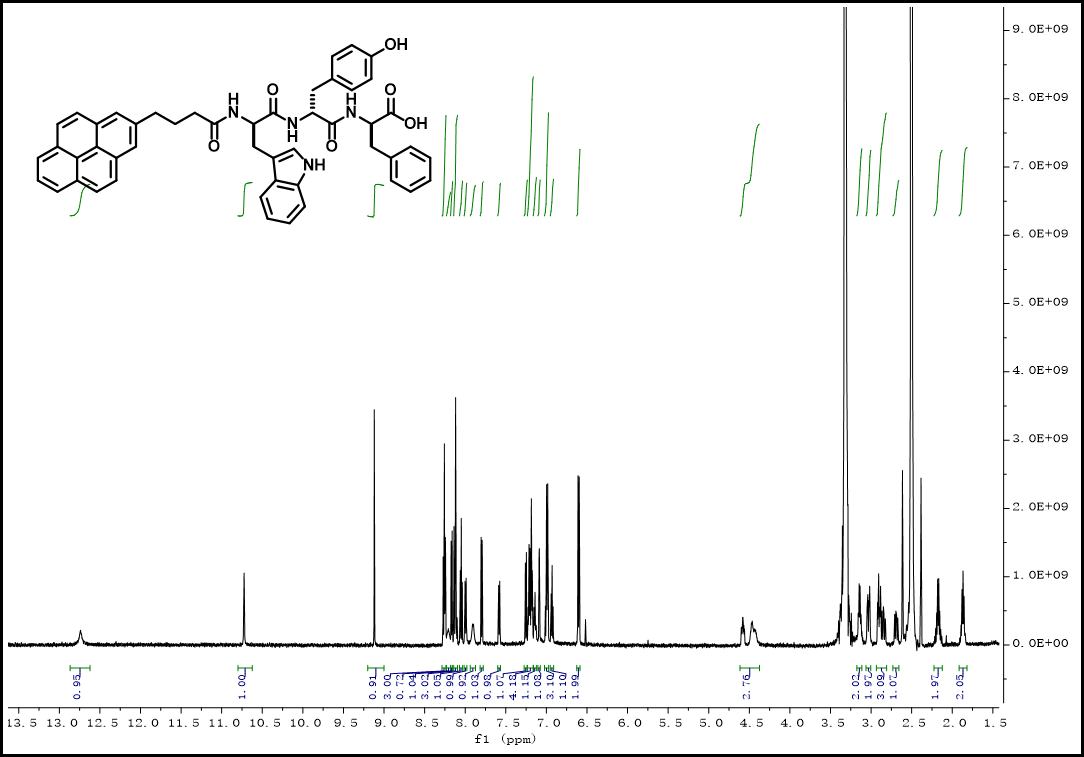


**Fig. S7.** ^1^H NMR spectrum of **LMW-D3**. δ12.75 (br, 1H, COOH), δ10.74 (s, 1H, NH in tryptophan), δ9.12 (s, 1H, OH in tyrosine), δ8.26 (m, 3H, NH), δ8.21-7.80 (m, 9H, CH in pyrene), δ7.59 (m, 1H, CH in tryptophan), δ7.26-6.93 (m, 11H, CH in tryptophan, tyrosine, and phenylalanine), δ6.60 (m, 2H, CH in tyrosine), δ4.58-4.46 (m, 3H, CH), δ3.15-2.68 (m, 8H, -CH_2_ in pyrene, tryptophan, phenylalanine, and tyrosine), δ2.21-1.87 (m, 4H, -CH_2_ in pyrene and tryptophan).


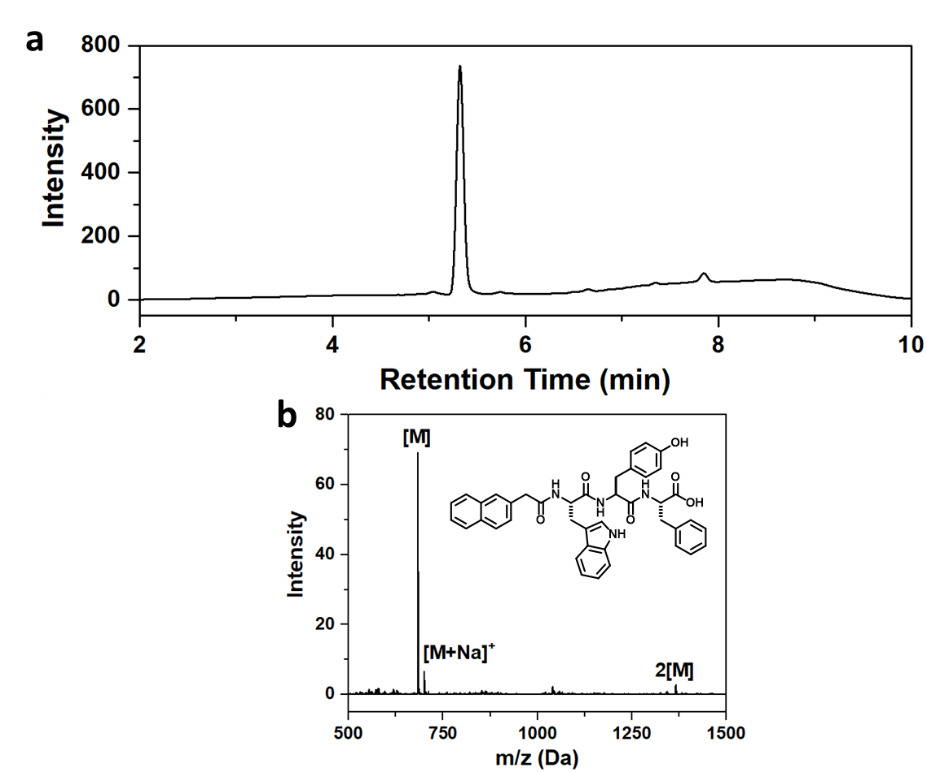


**Fig. S8.** LC-MS spectrum of **LMW-L1**. The retention time is 5.3 min, and the corresponding m/z is 683.7 Da, which is in accordance with theoretical molecular weight. The purity of **LMW-L1** is over 95%.


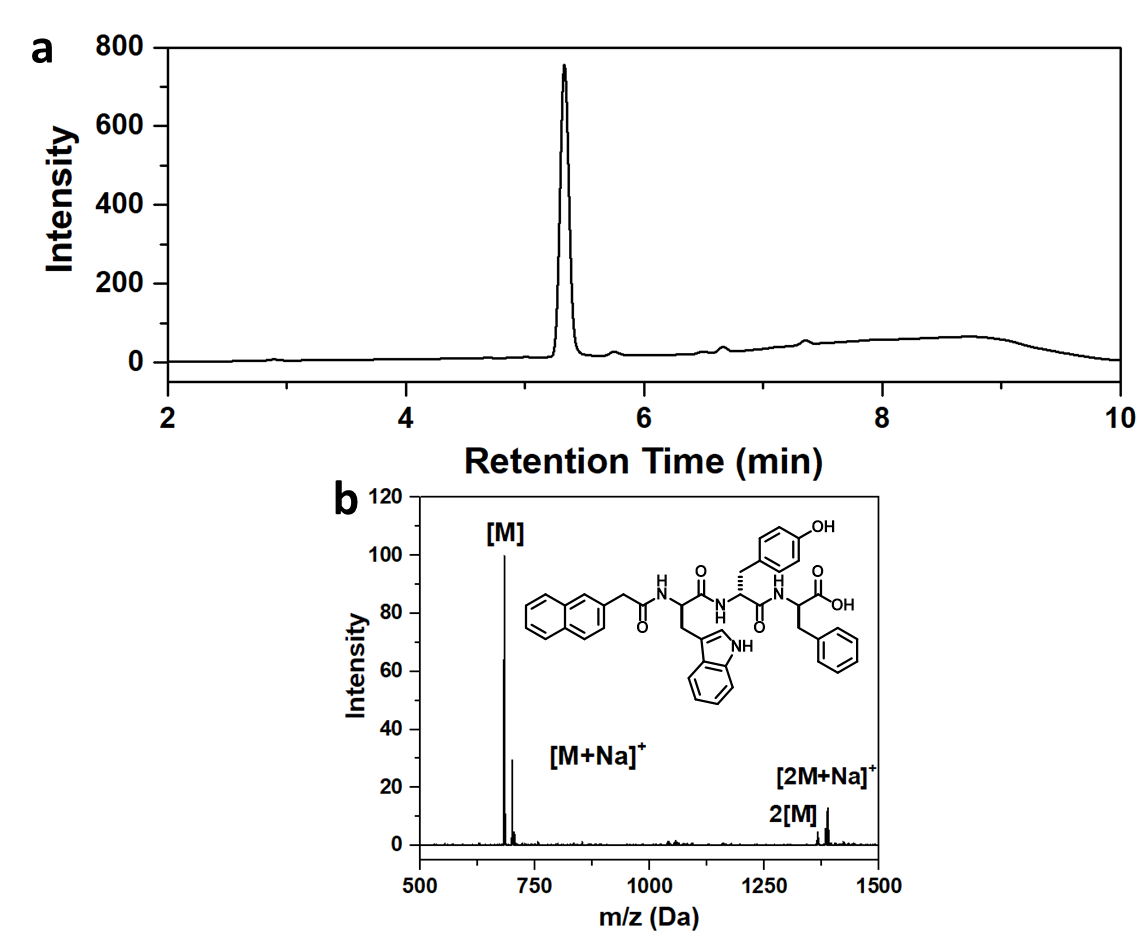


**Fig. S9.** LC-MS spectrum of **LMW-D1**. The retention time is 5.3 min, and the corresponding m/z is 683.7 Da, which is in accordance with theoretical molecular weight. The purity of **LMW-D1** is over 95%.


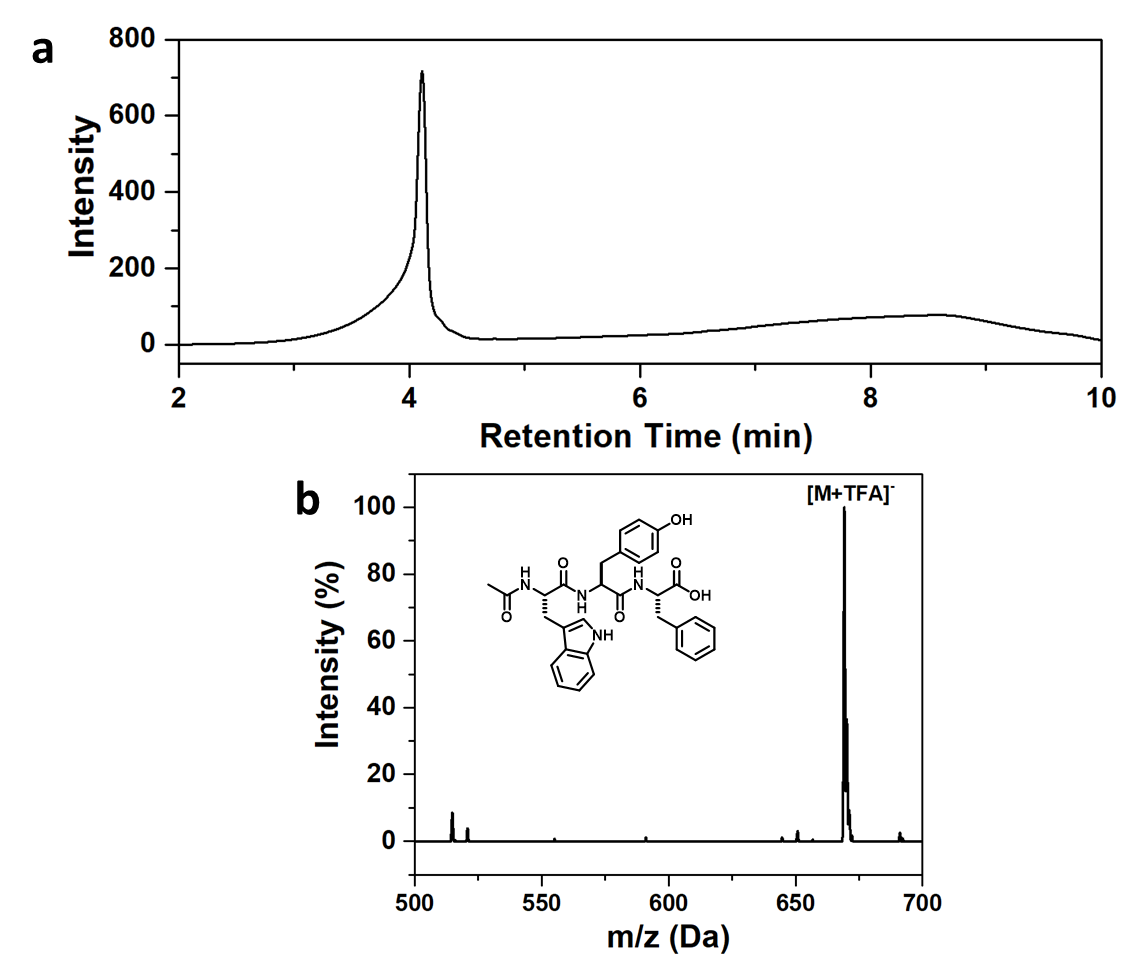


**Fig. S10.** LC-MS spectrum of **LMW-L2**. The retention time is 4.1 min, and the corresponding m/z is 556.2 Da, which is in accordance with theoretical molecular weight. The purity of **LMW-L2** is over 95%.


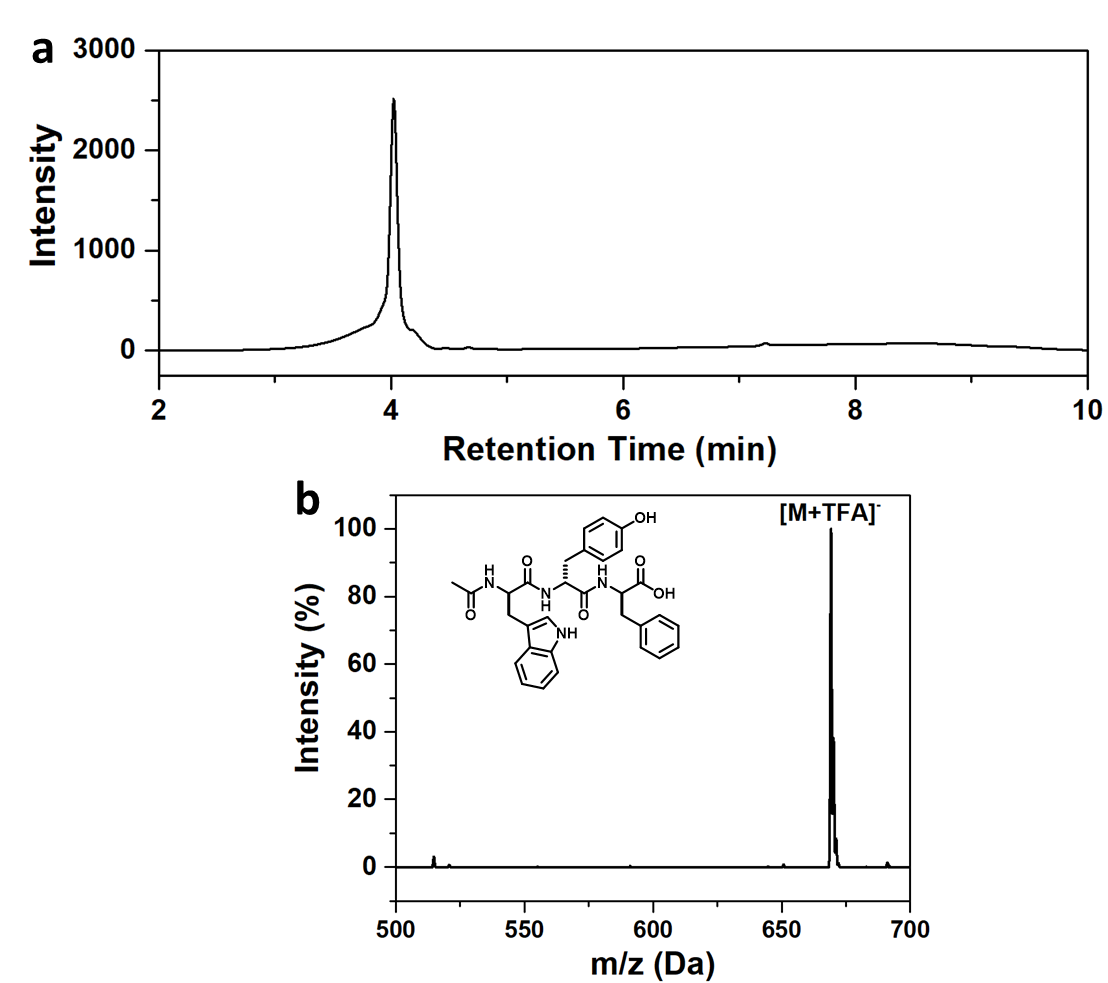


**Fig. S11.** LC-MS spectrum of **LMW-D2**. The retention time is 4.0 min, and the corresponding m/z is 556.2 Da, which is in accordance with theoretical molecular weight. The purity of **LMW-D2** is over 95%.


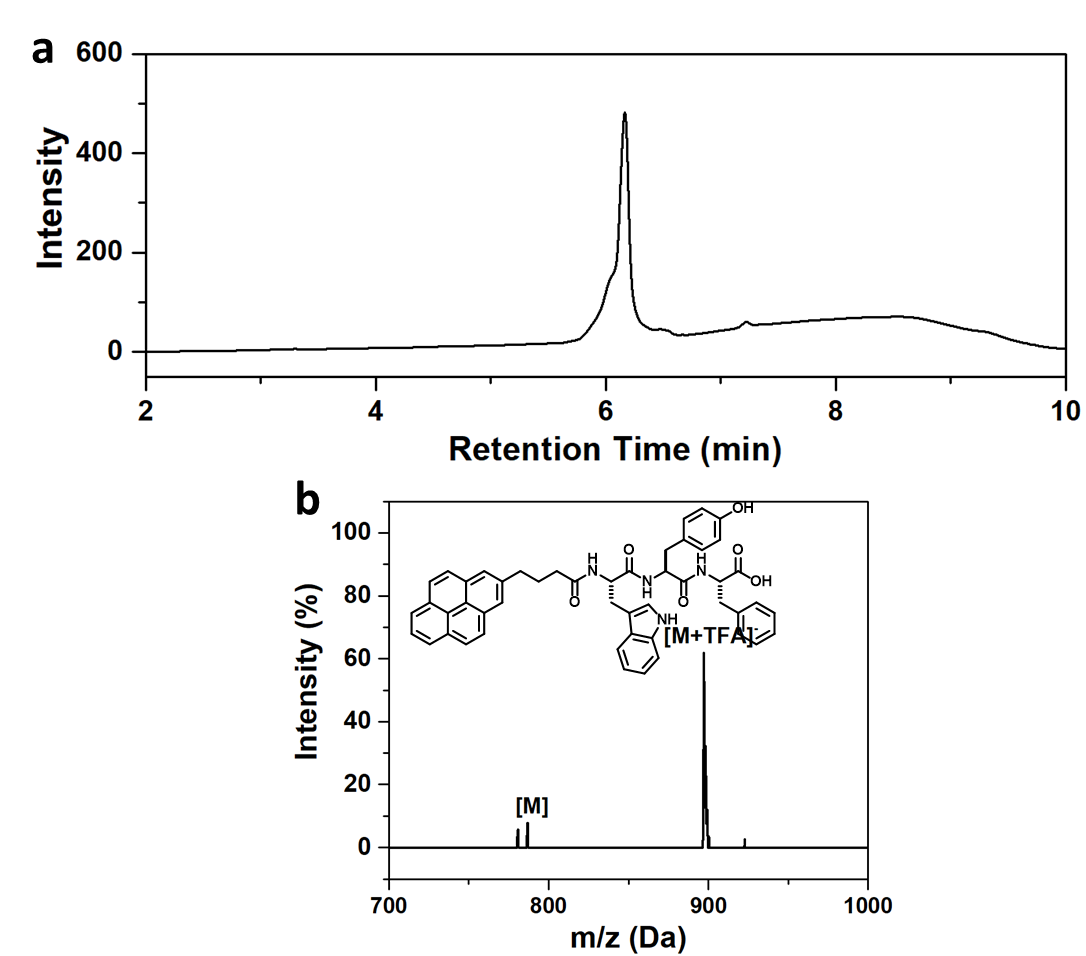


**Fig. S12.** LC-MS spectrum of **LMW-L3**. The retention time is 6.2 min, and the corresponding m/z is 784.9 Da, which is in accordance with theoretical molecular weight. The purity of **LMW-L3** is over 95%.


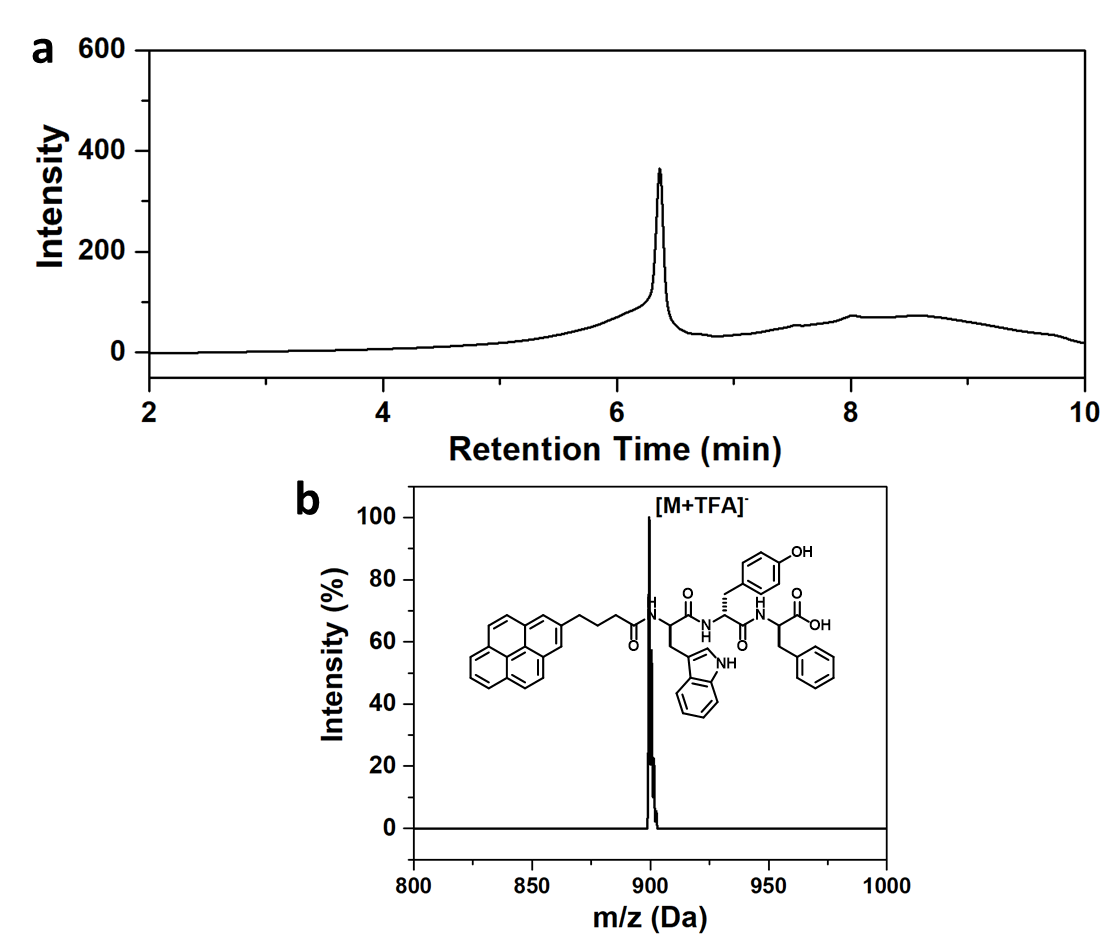


**Fig. S13.** LC-MS spectrum of **LMW-D3**. The retention time is 6.4 min, and the corresponding m/z is 784.9 Da, which is in accordance with theoretical molecular weight. The purity of **LMW-D3** is over 95%.


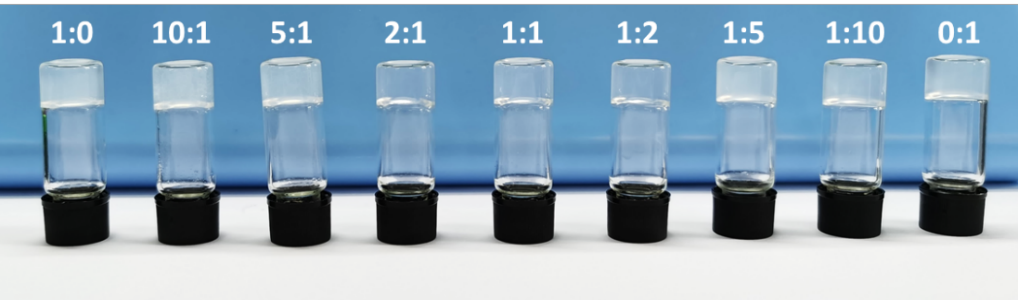


**Fig. S14.** Optical images of hydrogels formed by **LMW-L1** and **LMW-D1** at different molar ratio. From left to right, the molar ratio is 1:0, 10:1, 5:1, 2:1, 1:1, 1:2, 1:5, 1:10, and 0:1, respectively. The total concentration of **LMW-L1** and **LMW-D1** is 0.3 wt%.


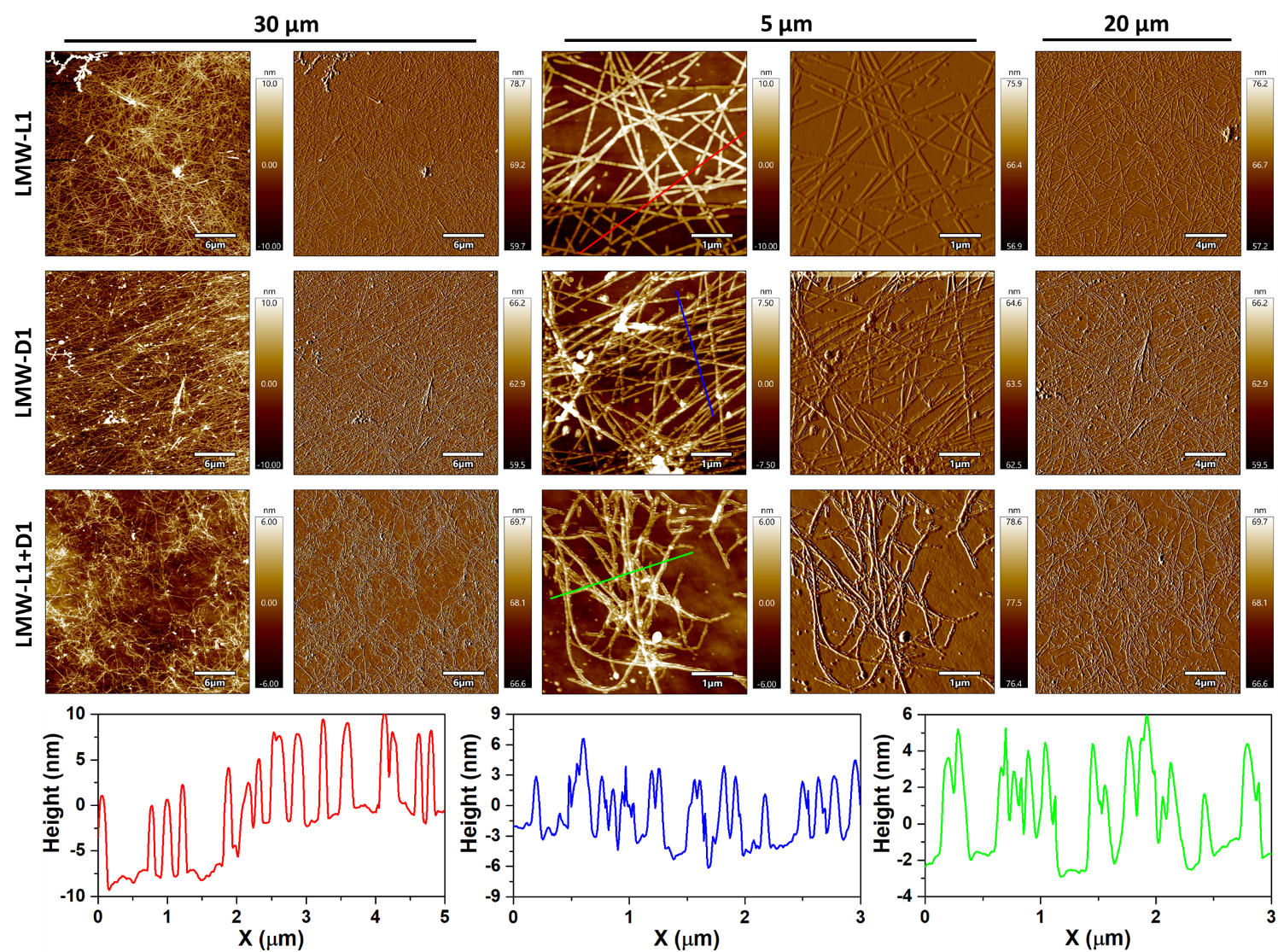


**Fig. S15.** AFM images and corresponding amplitude images of nanofibers formed by **LMW-L1** and **LMW-D1** at a molar ratio of 1:0, 0:1, and 1:1, respectively. The total concentration of **LMW-L1** and **LMW-D1** is 0.3 wt%.


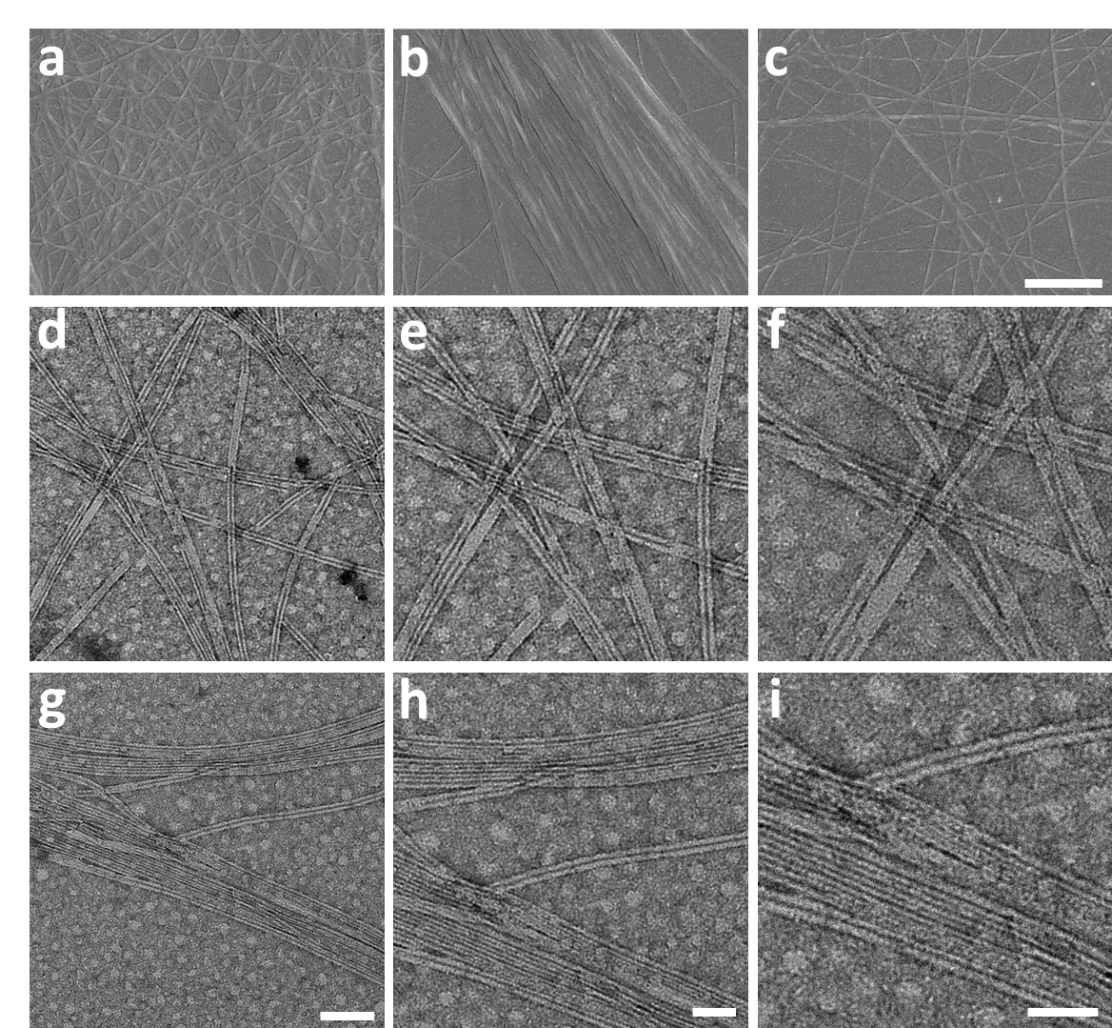


**Fig. S16.** a-c) SEM and d-i) high-magnification TEM images of nanofibers formed by **LMW-L1** and **LMW-D1** at a molar ratio of a, d-f) 1:0, b, g-i) 0:1, and c) 1:1, respectively. The total concentration of **LMW-L1** and **LMW-D1** is 0.3 wt%. Scale bar of a-c), d, g), e, h), and f, i) is 500 nm, 100 nm, 50 nm, and 50 nm, respectively.


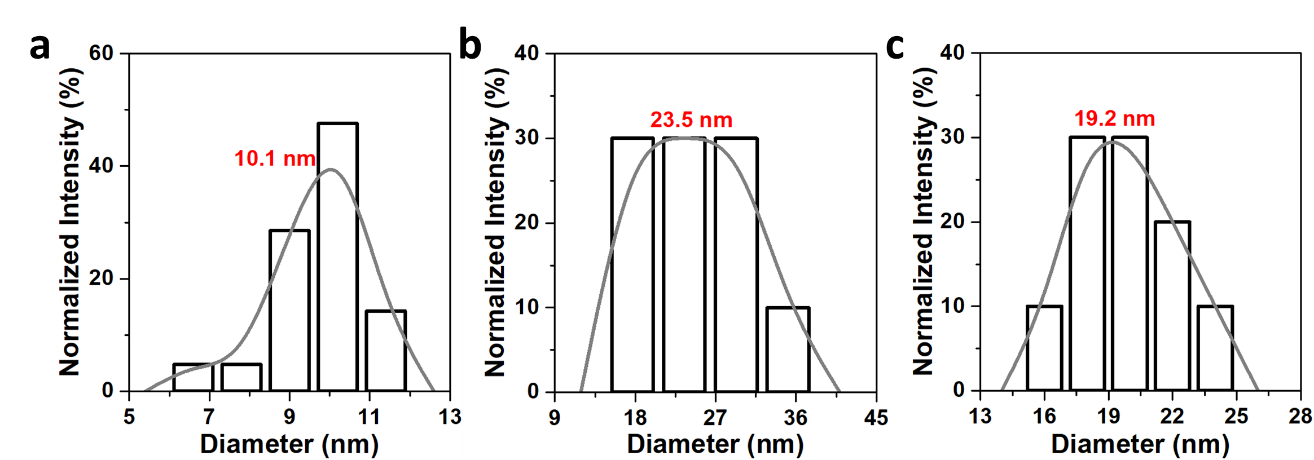


**Fig. S17.** Statistic diameter of nanofibers formed by **LMW-L1** and **LMW-D1** at a molar ratio of a) 1:0, b) 0:1, and c) 1:1, respectively. The total concentration of **LMW-L1** and **LMW-D1** is 0.3 wt%.


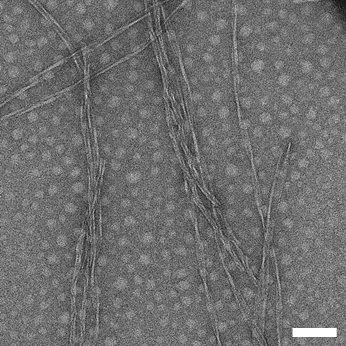


**Fig. S18.** TEM images of hydrogels formed by equimolar mixture of **LMW-L1** and **LMW-D1**. The total concentration of **LMW-L1** and **LMW-D1** is 0.3 wt%. Scale bar is 100 nm.


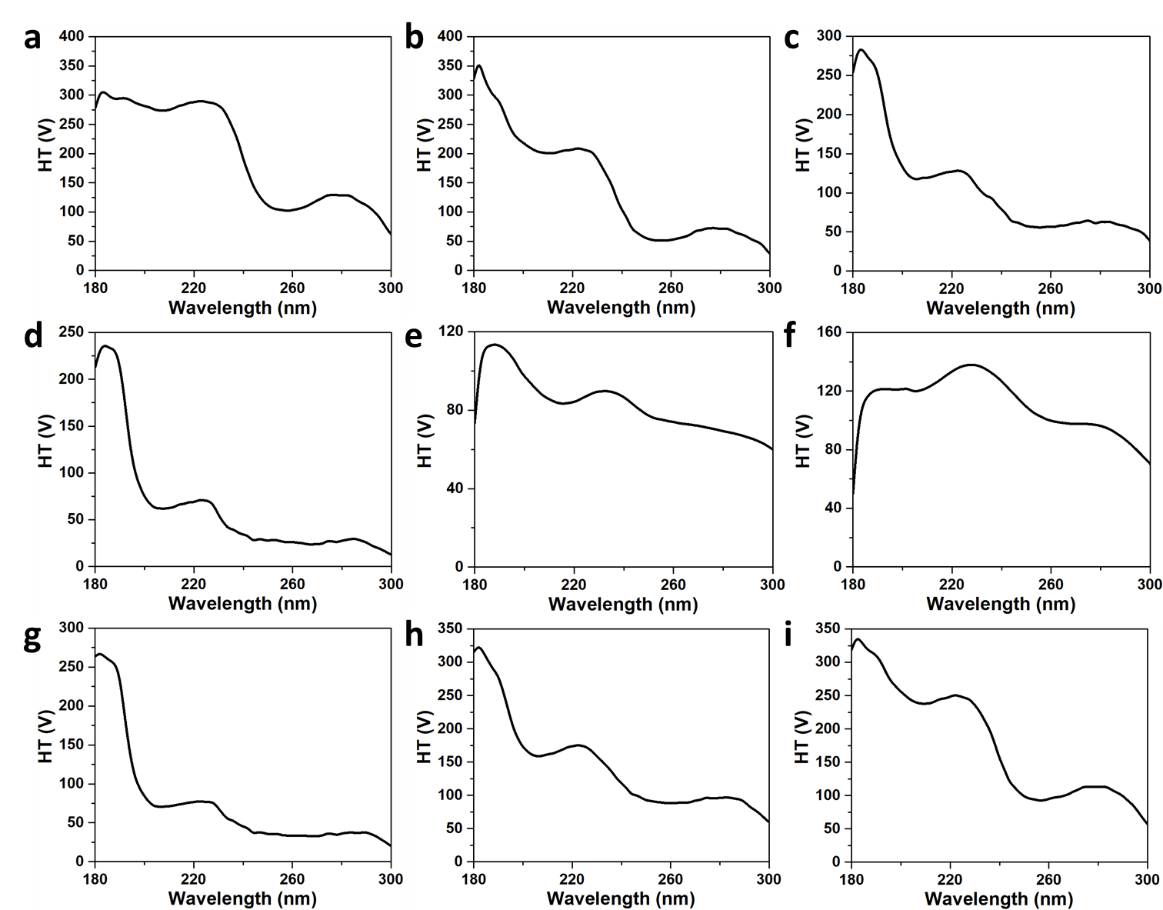


**Fig. S19.** HT data of hydrogels formed by **LMW-L1** and **LMW-D1** at a molar ratio of a) 1:0, b) 10:1, c) 5:1, d) 2:1, e) 1:1, f) 1:2, g) 1:5, h) 1:10, and i) 0:1. The HT trace is equivalent to the hydrogel’s absorbance. The total concentration of **LMW-L1** and **LMW-D1** is 0.3 wt%.


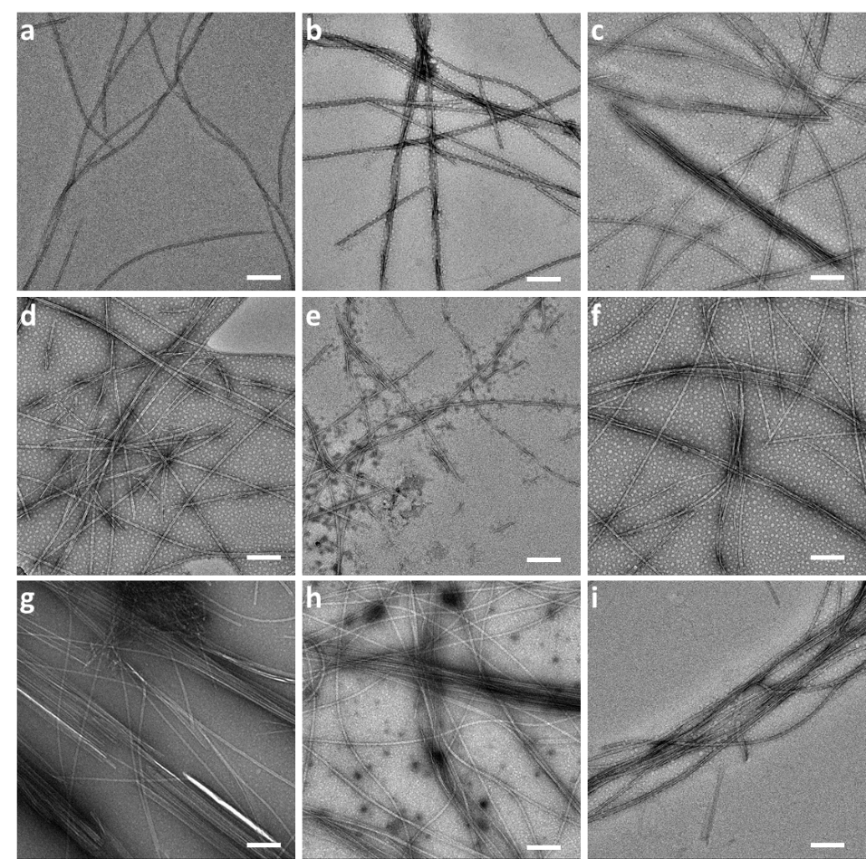


**Fig. S20.** TEM images of hydrogels formed by **LMW-L1** and **LMW-D1** at a molar ratio of a) 1:0, b) 10:1, c) 5:1, d) 2:1, e) 1:1, f) 1:2, g) 1:5, h) 1:10, and i) 0:1. Scale bar is 200 nm. The total concentration of **LMW-L1** and **LMW-D1** is 0.3 wt%.


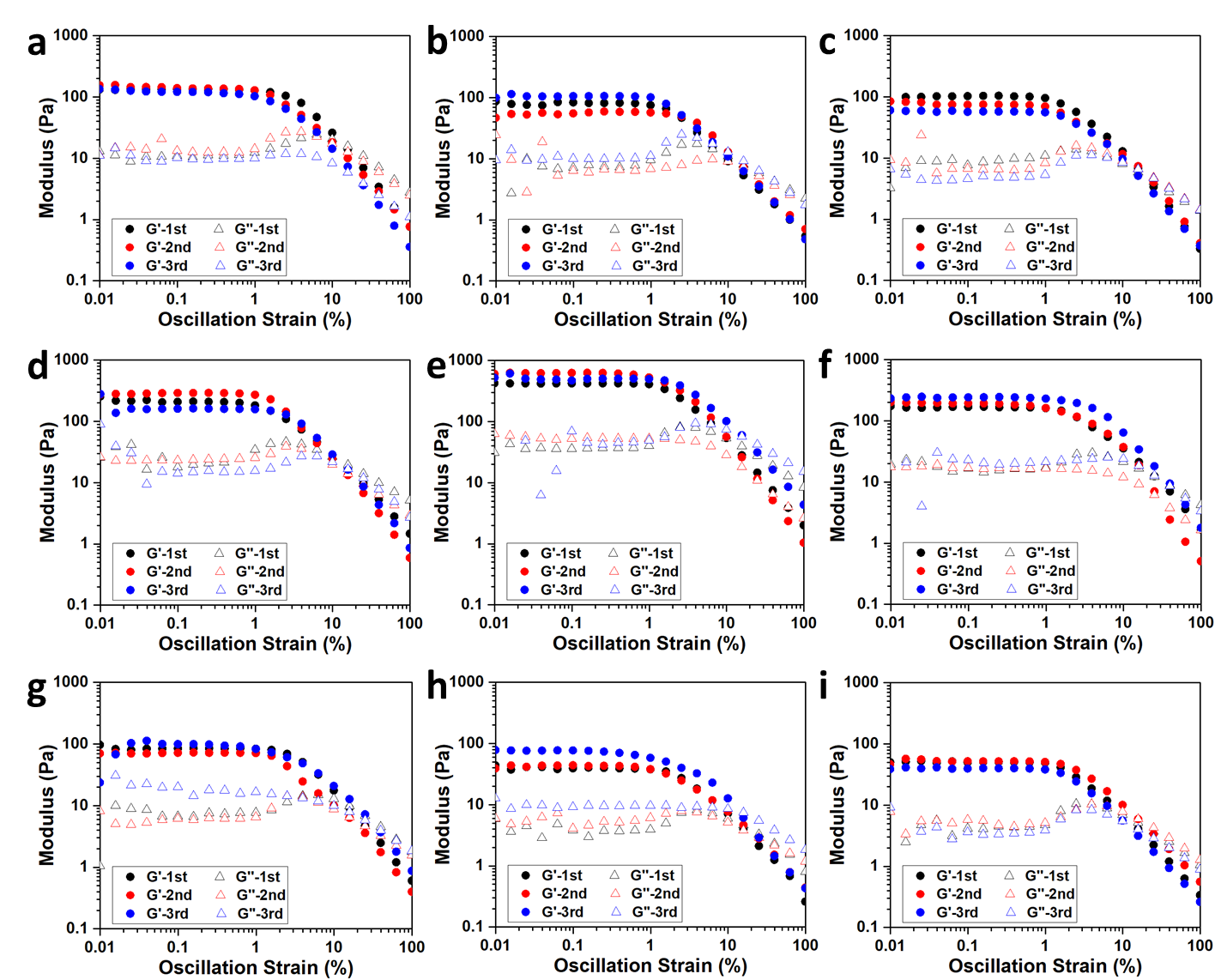


**Fig. S21.** Strain sweep (0.01% -100%) of hydrogels formed by **LMW-L1** and **LMW-D1** at a molar ratio of a) 1:0, b) 10:1, c) 5:1, d) 2:1, e) 1:1, f) 1:2, g) 1:5, h) 1:10, and i) 0:1, respectively. The total concentration of **LMW-L1** and **LMW-D1** is 0.3 wt%.


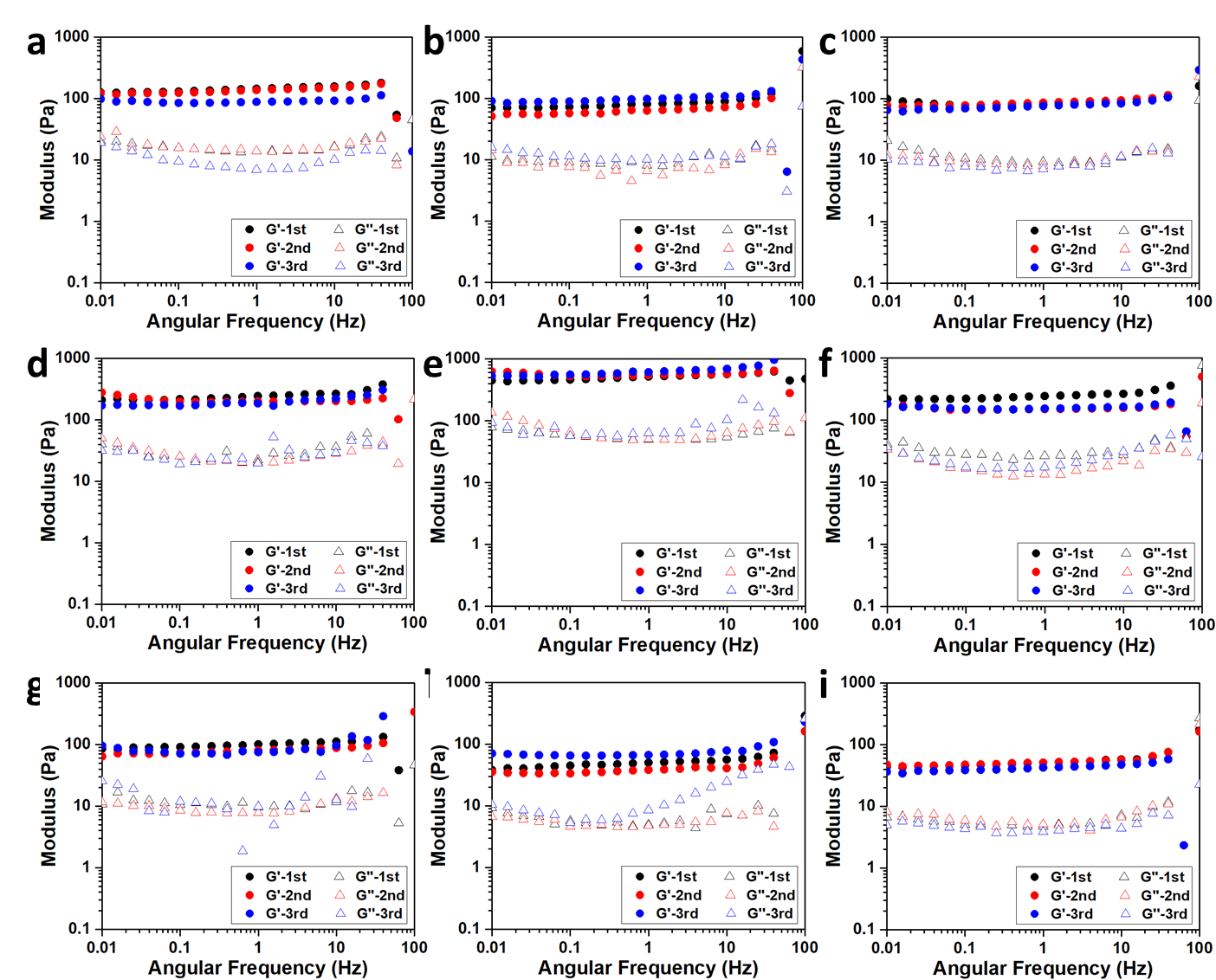


**Fig. S22.** Three individual results of frequency sweep (0.1-100 Hz) of hydrogels that formed by **LMW-L1** and **LMW-D1** at a molar ratio of a) 1:0, b) 10:1, c) 5:1, d) 2:1, e) 1:1, f) 1:2, g) 1:5, h) 1:10, and i) 0:1, respectively. The fixed strain is 0.5%. The total concentration of **LMW-L1** and **LMW-D1** is 0.3 wt%.


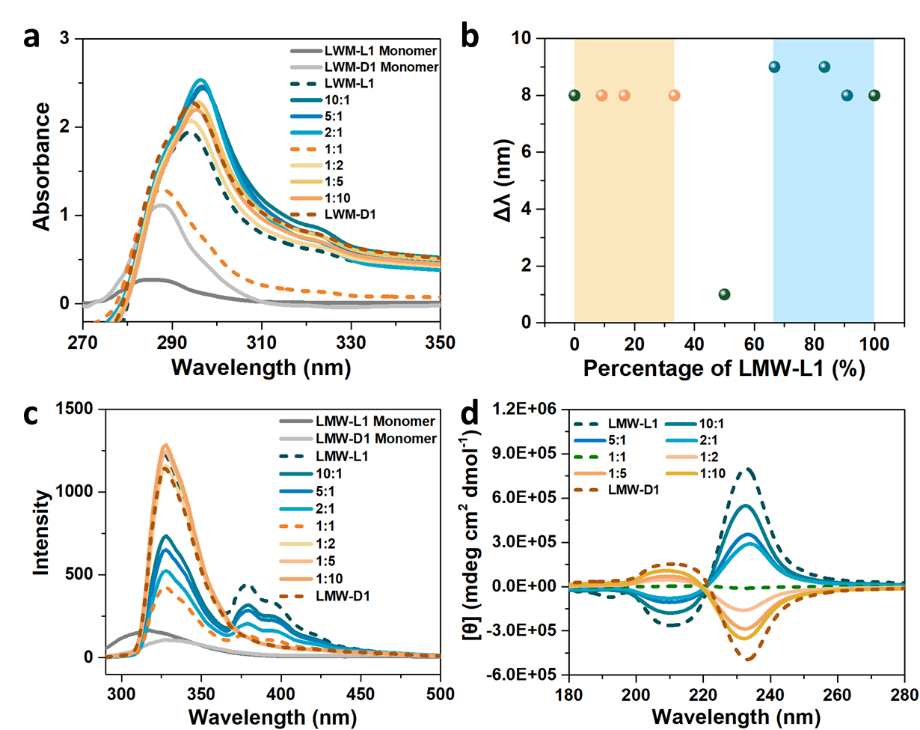


**Fig. S23.** a) UV-vis spectra, b) the wavelength offset in UV-vis spectra, c) fluorescence spectra, and d) CD spectra, of hydrogels formed by **LMW-L1** and **LMW-D1** at different molar ratio. The total concentration of **LMW-L1** and **LMW-D1** is 0.3 wt%.


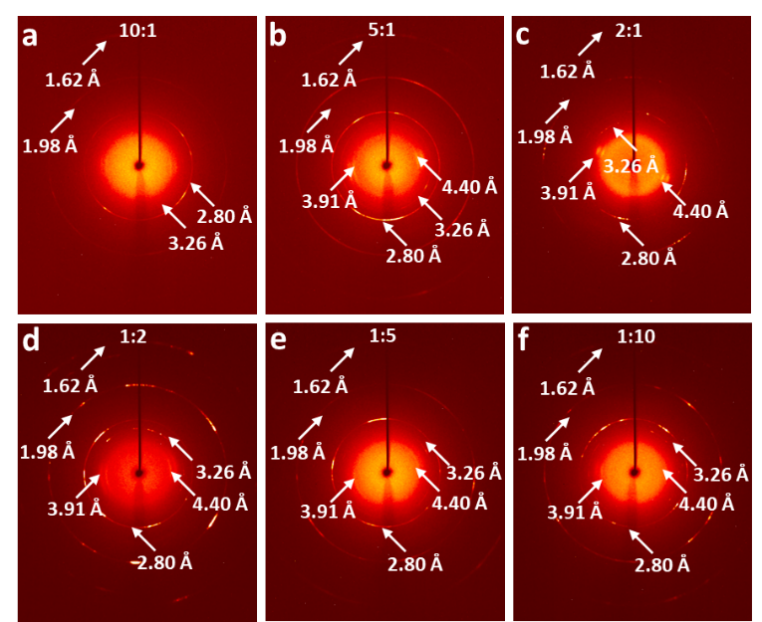


**Fig. S24.** *In situ* WAXS patterns of hydrogels formed by **LMW-L1** and **LMW-D1** with a molar ratio of a) 10:1, b) 5:1, c) 2:1, d) 1:2, e) 1:5, and f) 1:10, respectively. The total concentration of **LMW-L1** and **LMW-D1** is 0.3 wt%.


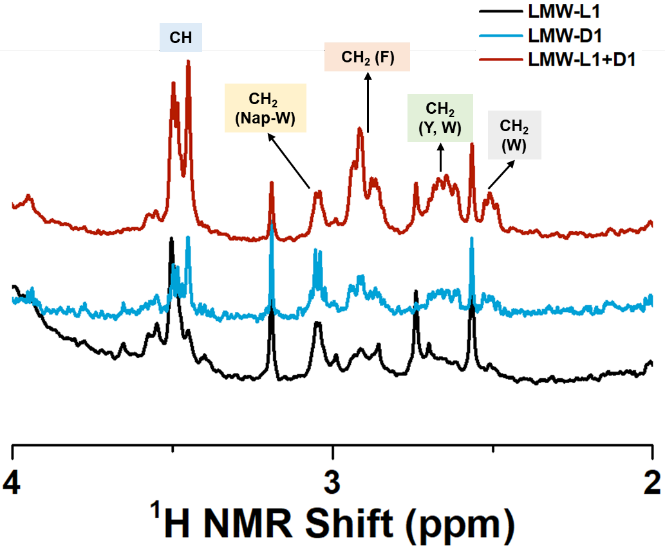


**Fig. S25.** ^1^H NMR spectrum of nanostructures formed by **LMW-L1** and **LMW-D1** in deuterated PBS at different molar ratio. The total concentration of **LMW-L1** and **LMW-D1** is 0.1 wt%.


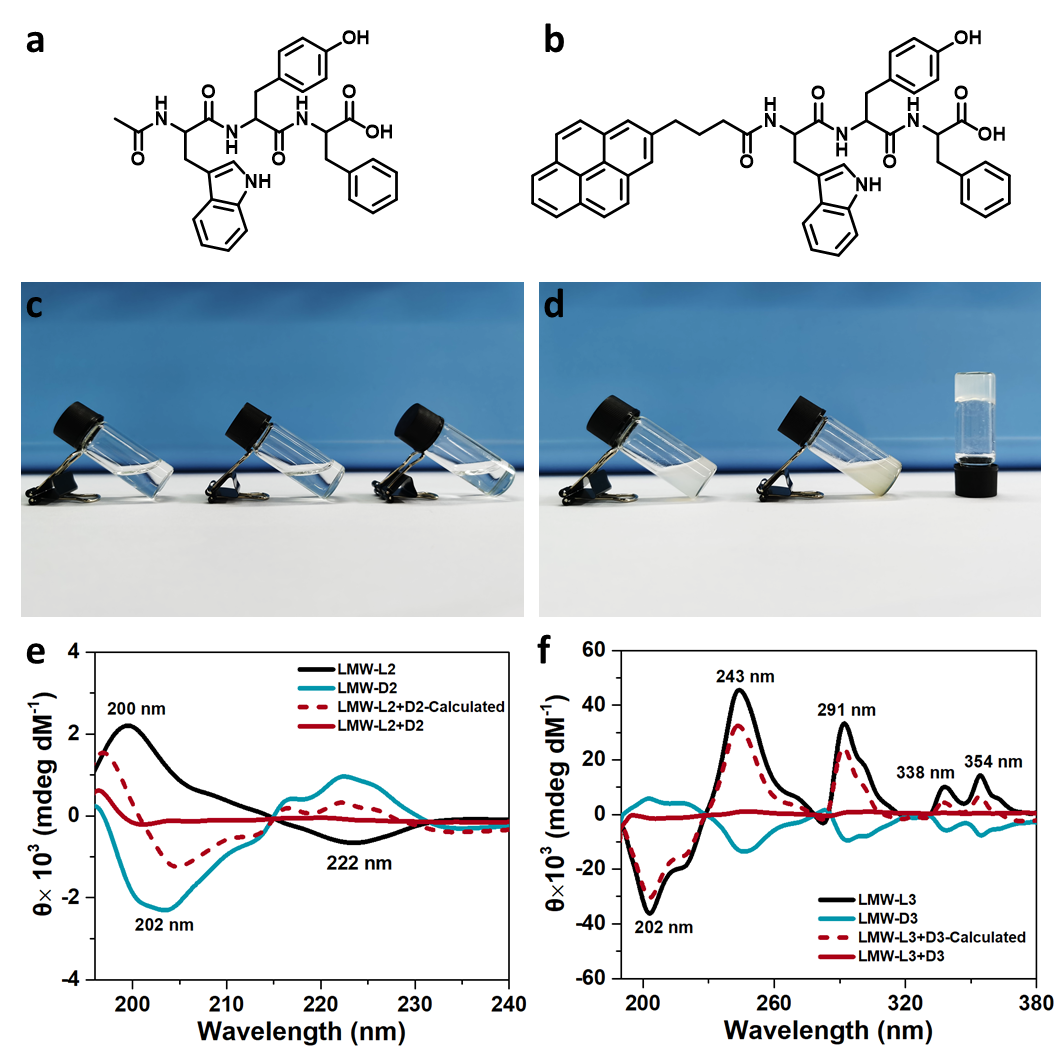


**Fig. S26.** Molecular structure of a) **LMW-2**, b) **LMW-3**, c, d) optical images and e, f) CD spectra of nanostructures formed by a, c) **LMW-L2** and **LMW-D2**, b, d) **LMW-L3** and **LMW-D3**. In the optical photographs, from left to right, the molar ratio is 1:0, 0:1 and 1:1, respectively. The total concentration of **LMW-L2** and **LMW-D2**, **LMW-L3** and **LMW-D3** is 0.3 wt%.


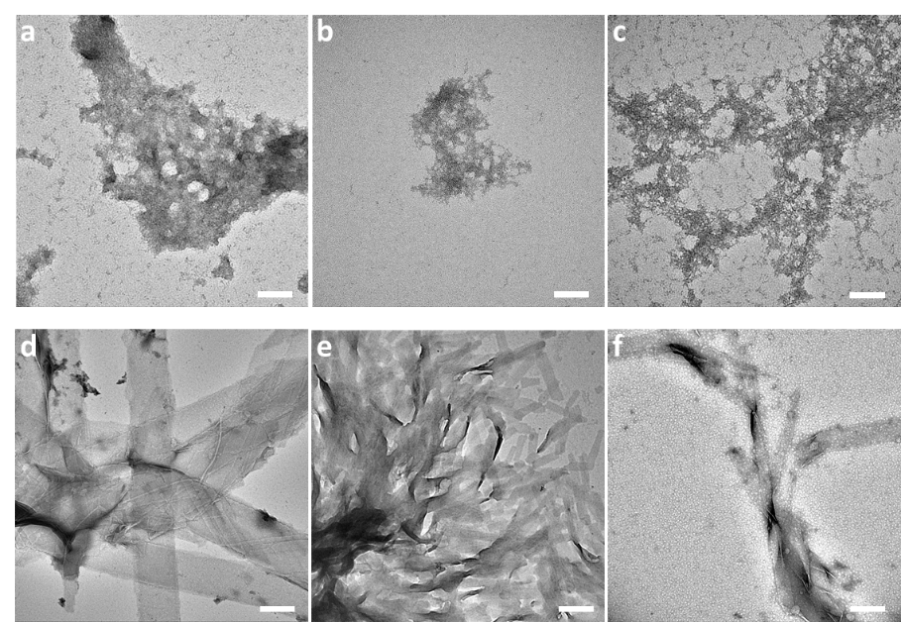


**Fig. S27.** TEM images of nanostructures formed by a-c) **LMW-L2** and **LMW-D2**, d-f) **LMW-L3** and **LMW-D3**, from left to right, the molar ratio is 1:0, 0:1 and 1:1, respectively. The scale bar for a-c) and d-f) is 50 nm and 200 nm, respectively. The total concentration of **LMW-L2** and **LMW-D2**, **LMW-L3** and **LMW-D3** is 0.3 wt%.


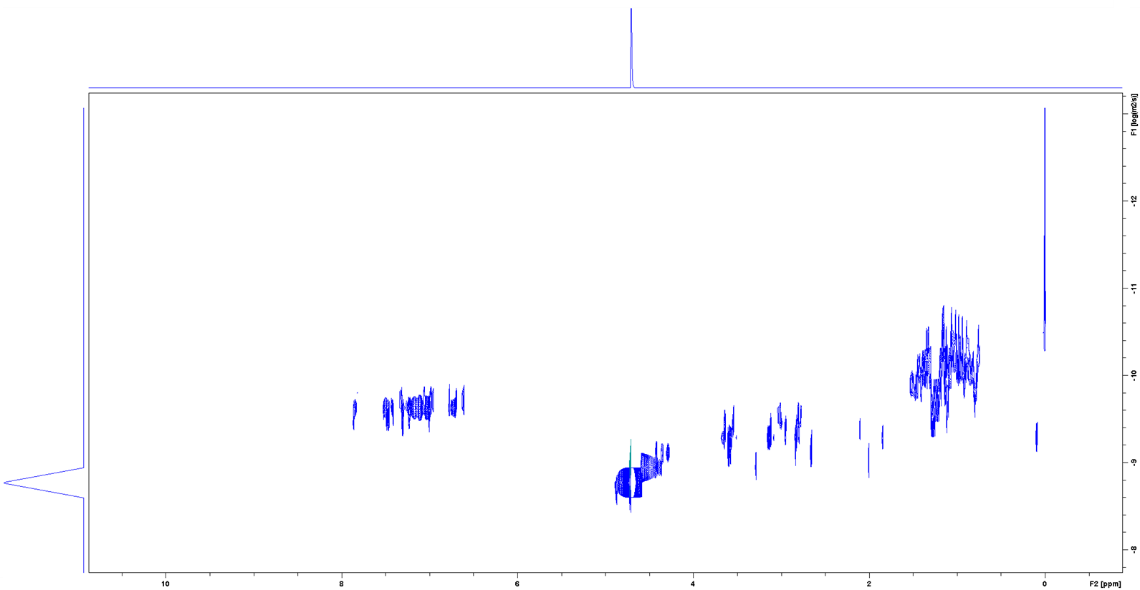


**Fig. S28.** 2D DOSY spectrum of nanostructures formed by **LMW-L1** in deuterated PBS at a concentration of 0.1 wt%. The lg D was measured as -9.655 m^2^ s^-1^.


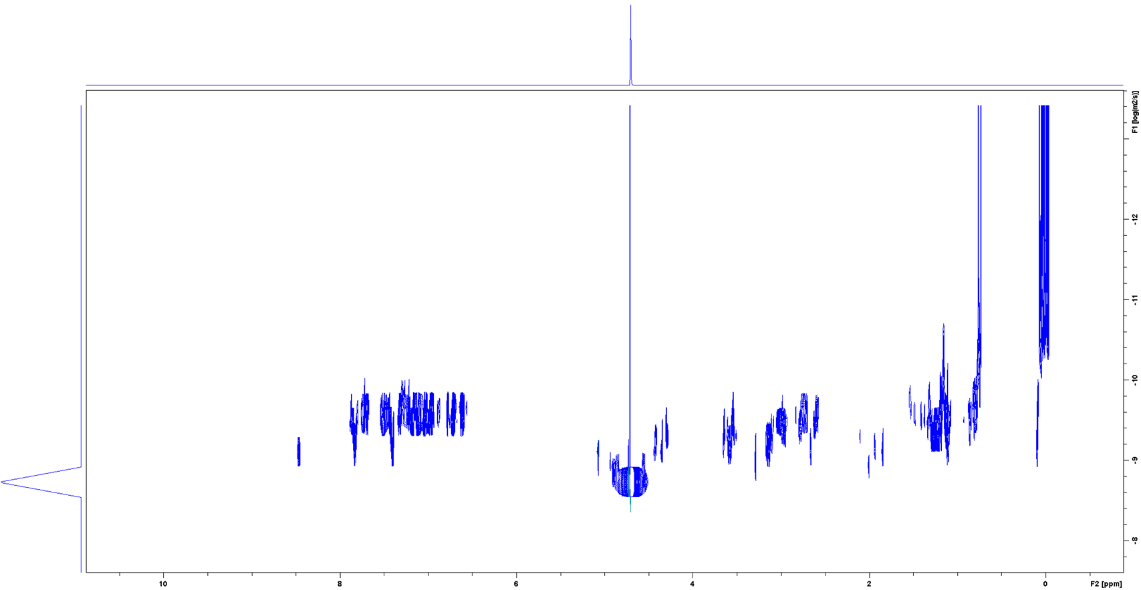


**Fig. S29.** 2D DOSY spectrum of nanostructures formed by **LMW-D1** in deuterated PBS at a concentration of 0.1 wt%. The lg D was measured as -9.597 m^2^ s^-1^.


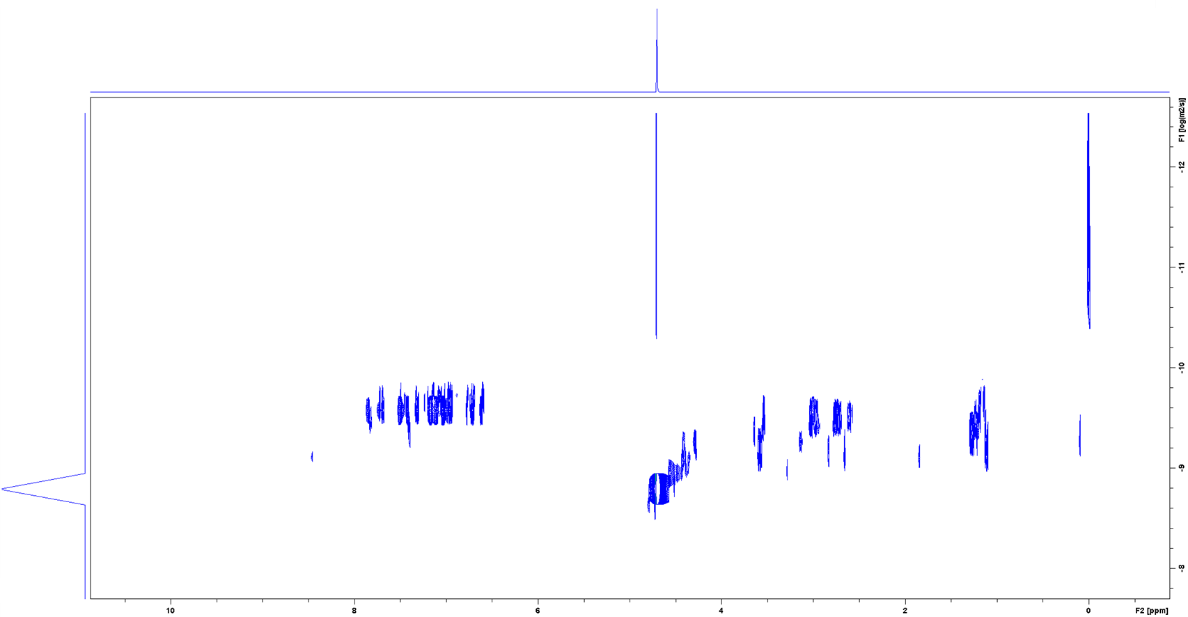


**Fig. S30.** 2D DOSY spectrum of nanostructures formed by equimolar ratio of **LMW-L1** and **LMW-D1** in deuterated PBS at a concentration of 0.1 wt%. The lg D was measured as -9.643 m^2^ s^-1^.


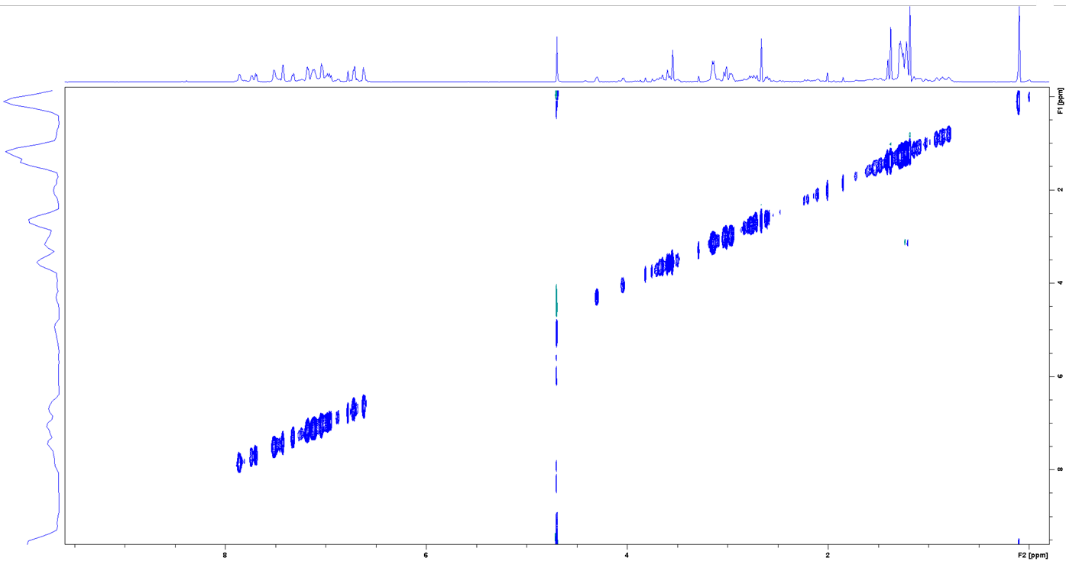


**Fig. S31.** 2D NOESY spectrum of nanostructures formed by **LMW-L1** in deuterated PBS at a concentration of 0.1 wt%.


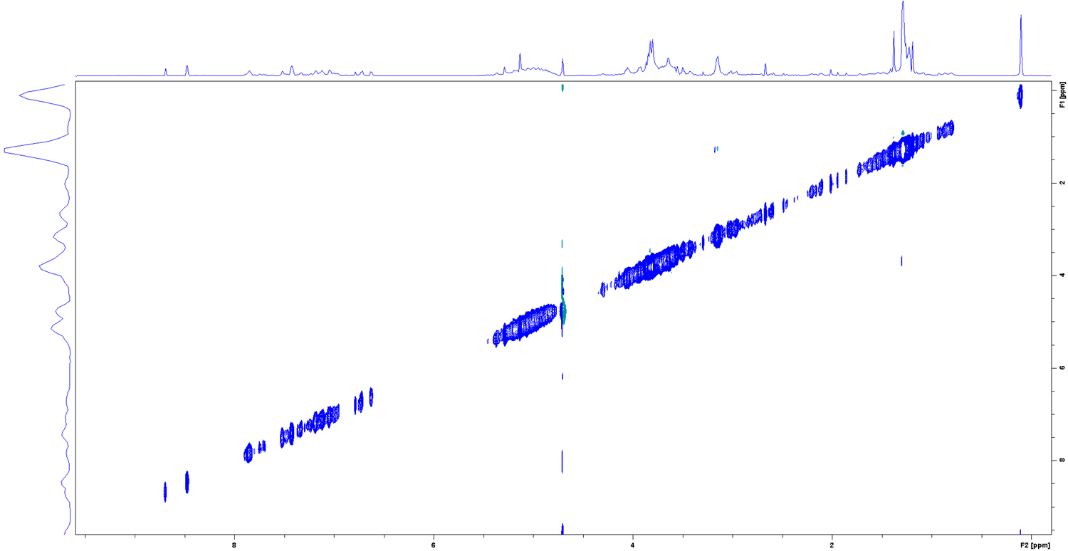


**Fig. S32.** 2D NOESY spectrum of nanostructures formed by **LMW-D1** in deuterated PBS at a concentration of 0.1 wt%.


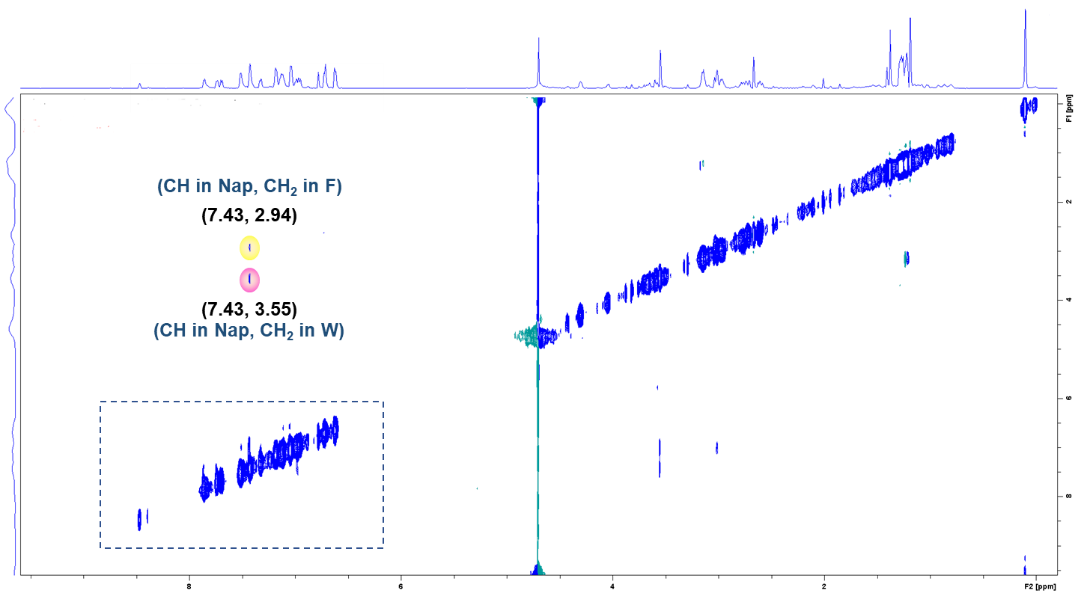


**Fig. S33.** 2D NOESY spectrum of nanostructures formed by equal molar of **LMW-L1** and **LMW-D1** in deuterated PBS at a total concentration of 0.1 wt%. Close contacts are showed with colored circles.


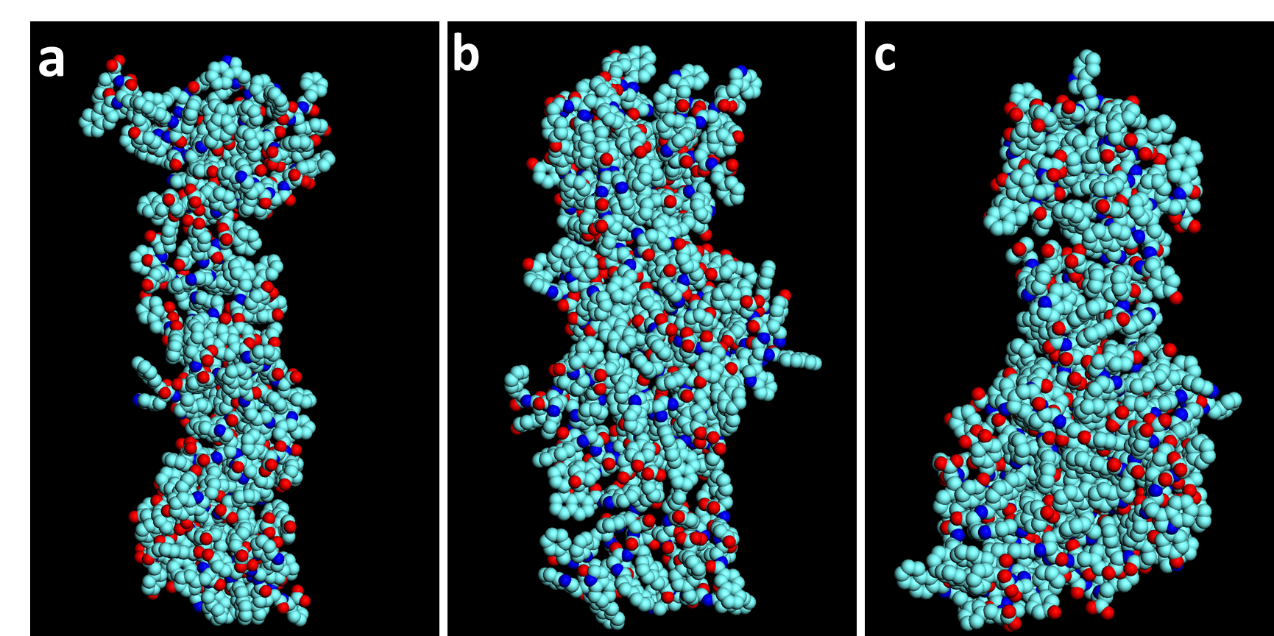


**Fig. S34.** Optimized structures of aggregates formed by **LMW-L1** and **LMW-D1** at a molar ratio of a) 1:0, b) 0:1, and c) 1:1 *via* MD calculations, respectively. The cyan, red, and blue ball represents the carbon, oxygen, and nitrogen, respectively.


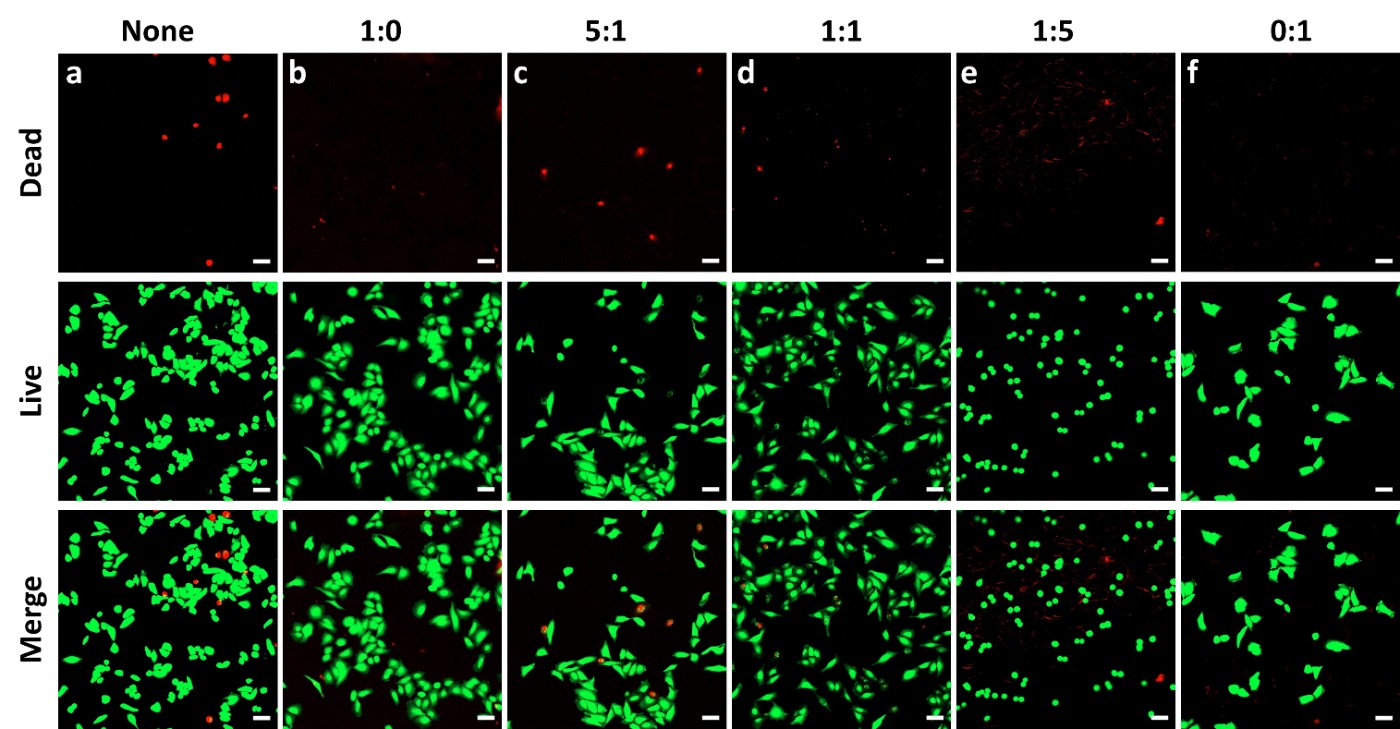


**Fig. S35.** Morphologies of HeLa cells incubated with a) culture medium and hydrogels formed by **LMW-L1** and **LMW-D1** at a molar ratio of b) 1:0, c) 5:1, d) 1:1, e) 1:5, and f) 0:1 after 24 h of live/dead test. The red and green fluorescence indicate the dead and live cells, respectively. Scale bar is 50 μm.


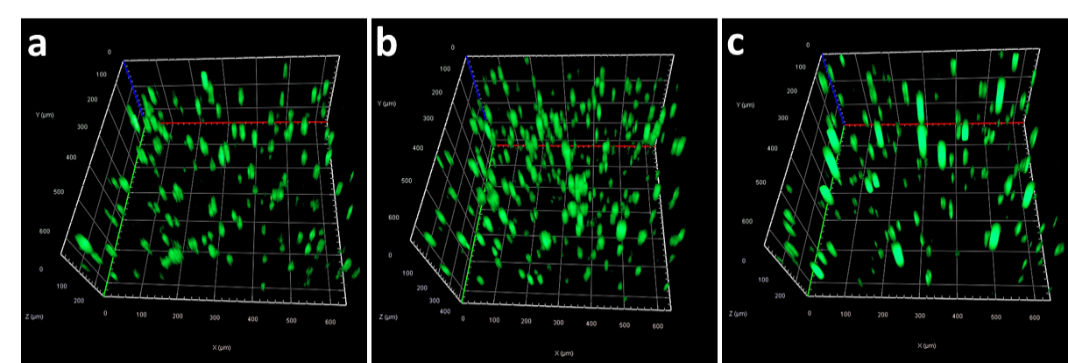


**Fig. S36.** 3D cell culture of HeLa cells in the hydrogels co-assembled by **LMW-L1** and **LMW-D1** at a molar ratio of a) 1:0, b) 1:1, and c) 0:1.


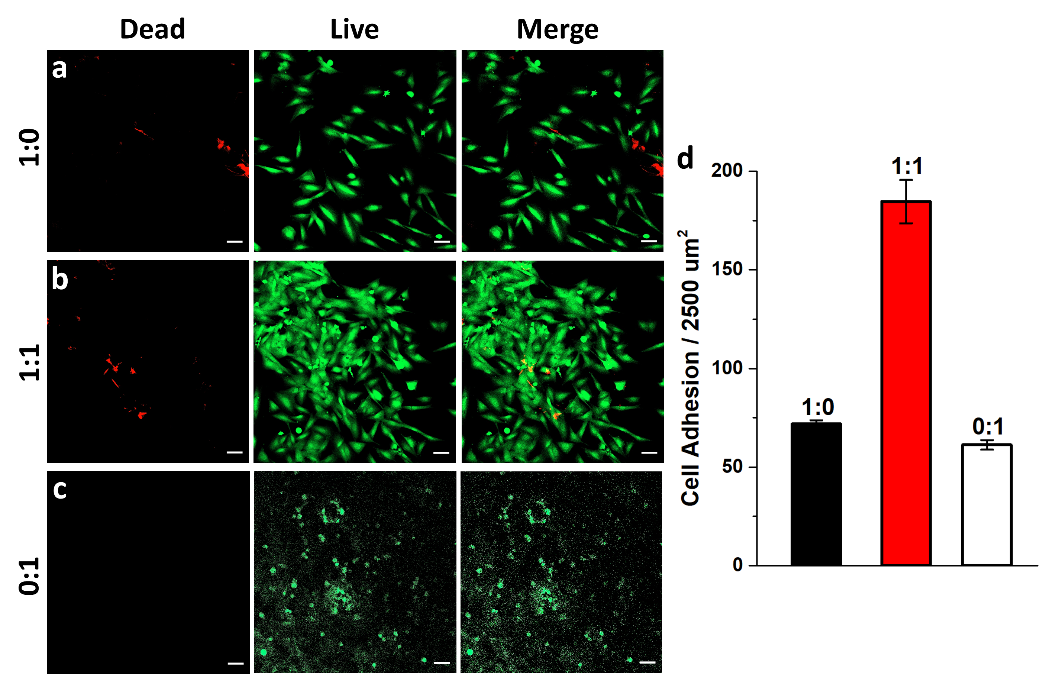


**Figure S37.** Morphologies of Saos-2 cells incubated with hydrogels formed by **LMW-L1** and **LMW-D1** at a molar ratio of a) 1:0, b) 1:1, and c) 0:1 after 24 h of live/dead test. d) The statistic adhesive Saos-2 cells on hydrogels formed by **LMW-L1** and **LMW-D1** at different molar ratios. The red and green fluorescence indicate the dead and live cells, respectively. Scale bar is 50 μm.


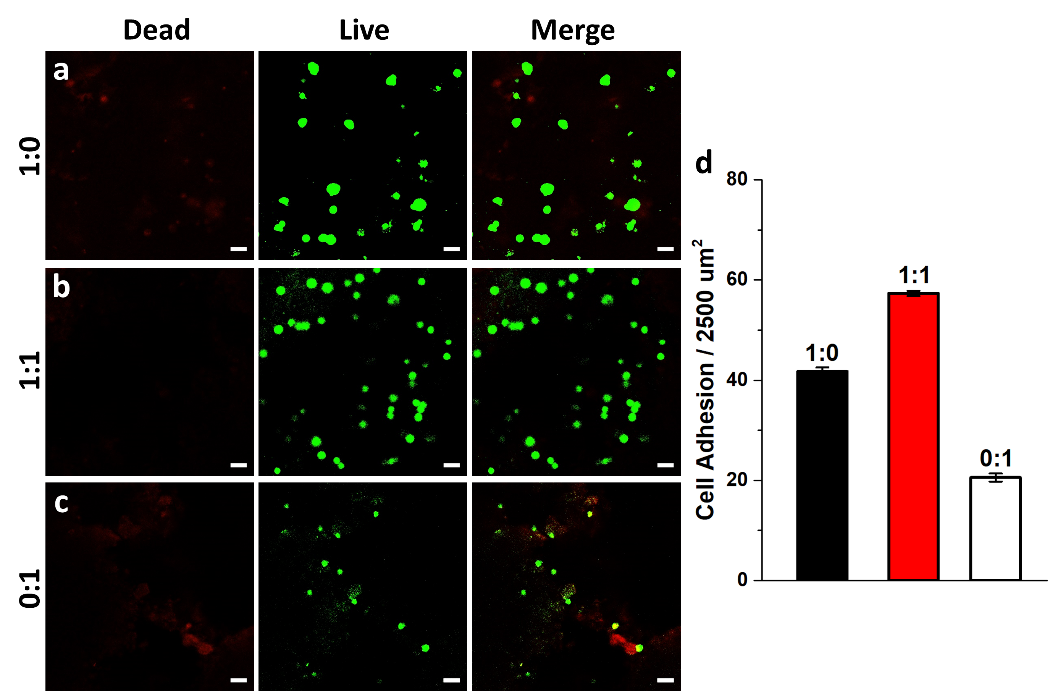


**Figure S38.** Morphologies of Neuro-2a cells incubated with hydrogels formed by **LMW-L1** and LMW-D1 at a molar ratio of a) 1:0, b) 1:1, and c) 0:1 after 24 h of live/dead test. d) The statistic adhesive Neuro-2a cells on hydrogels formed by **LMW-L1** and **LMW-D1** at different molar ratios. The red and green fluorescence indicate the dead and live cells, respectively. Scale bar is 50 μm.


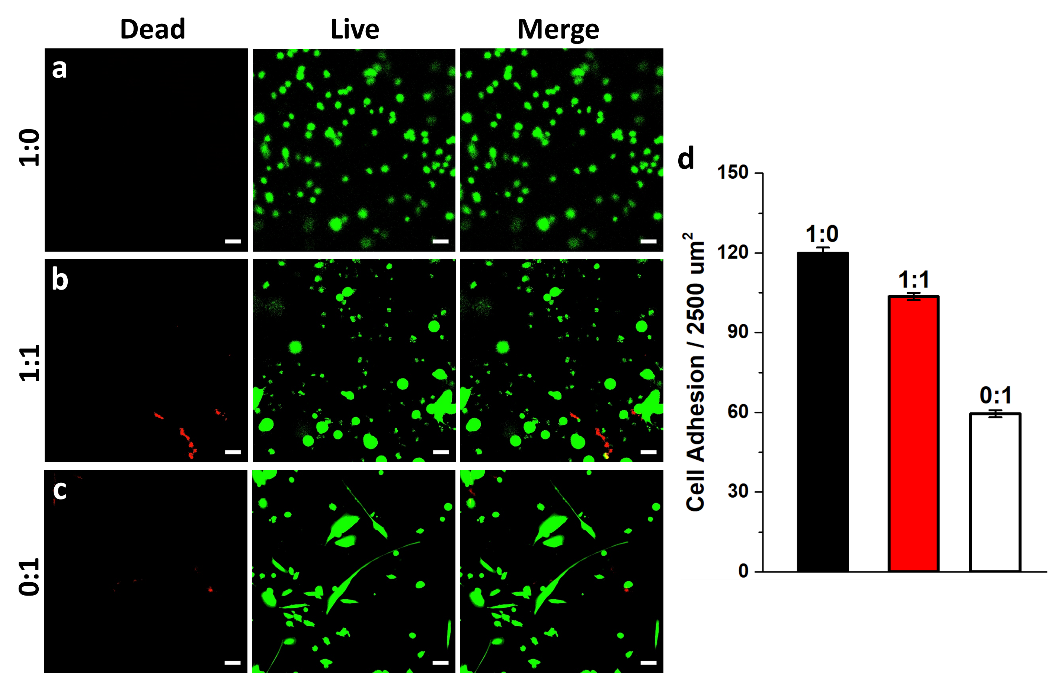


**Figure S39.** Morphologies of HS-5 cells incubated with hydrogels formed by **LMW-L1** and **LMW-D1** at a molar ratio of a) 1:0, b) 1:1, and c) 0:1 after 24 h of live/dead test. d) The statistic adhesive HS-5 cells on hydrogels formed by **LMW-L1** and **LMW-D1** at different molar ratios. The red and green fluorescence indicate the dead and live cells, respectively. Scale bar is 50 μm.

**S5. Supporting tables**

**Table S1.** LCM-MS method.

| **Time (min)** | **A-H_2_O (%)** | **B-CH_3_CN (%)** | **Flow (mL/min)** |
| --- | --- | --- | --- |
| 0 | 90 | 10 | 0.6 |
| 5 | 30 | 70 | 0.6 |
| 5.5 | 0 | 100 | 0.6 |
| 7 | 0 | 100 | 0.6 |
| 8 | 90 | 10 | 0.6 |
| 10 | 90 | 10 | 0.6 |

**Table S2.** The gelation time of different hydrogels co-assembled by **LMW-L1** and **LMW-D1**.

| Molar Ratio (**LMW-L1**:**D1**) | Gelation Time (min) |
| --- | --- |
| 1:0 | 5-10 |
| 10:1 | 5-10 |
| 5:1 | <5 |
| 2:1 | <5 |
| 1:1 | <5 |
| 1:2 | <5 |
| 1:5 | <5 |
| 1:10 | 5-10 |
| 0:1 | 5-10 |

**Table S3.** Storage modulus and loss modulus in stress-strain and frequency sweep experiment of hydrogels formed by **LMW-L1** and **LMW-D1** at various molar ratio. The fixed strain in frequency sweep experiment is 0.5%. The total concentration of **LMW-L1** and **LMW-D1** is 0.3 wt%.

| Molar Ratio (**LMW-L1**: **LMW-D1**) | Stable  Angular  Frequency (Hz) | Storage  Modulus  (Pa) | Loss  Modulus  (Pa) | Linear  viscoelastic  region  (%) | Storage  Modulus  (Pa) | Loss  Modulus  (Pa) |
| --- | --- | --- | --- | --- | --- | --- |
| 1:0 | 0.01-38 | 124.7±28.4 | 12.6±2.2 | 0.01-2.23 | 126.6±13.1 | 11.2±1.4 |
| 10:1 | 0.01-36 | 80.7±17.6 | 8.2±1.8 | 0.01-1.17 | 87.6±23.3 | 8.1±1.9 |
| 5:1 | 0.01-42 | 81.6±5.1 | 8.2±1.1 | 0.01-1.21 | 77.5±22.4 | 7.2±2.5 |
| 2:1 | 0.01-37 | 215.7±30.3 | 26.6±5.2 | 0.01-1.66 | 216.1±64.2 | 21.4±5.5 |
| 1:1 | 0.01-40 | 558.4±46.9 | 54.1±7.8 | 0.01-2.71 | 552.6±91.6 | 49.6±8.5 |
| 1:2 | 0.01-34 | 182.4±53.2 | 19.2±6.7 | 0.01-2.39 | 194.3±39.1 | 17.8±2.4 |
| 1:5 | 0.01-26 | 85.1±10.6 | 7.0±4.8 | 0.01-1.98 | 82.9±10.0 | 9.8±5.2 |
| 1:10 | 0.01-26 | 51.6±14.1 | 5.7±1.5 | 0.01-1.47 | 48.6±14.4 | 6.3±3.0 |
| 0:1 | 0.01-37 | 48.2±2.0 | 4.5±0.6 | 0.01-2.01 | 47.3±6.7 | 4.3±0.7 |
